# Amine Containing Analogs of Sulindac for Cancer Prevention

**DOI:** 10.2174/1874104501812010001

**Published:** 2018-01-31

**Authors:** Bini Mathew, Judith V. Hobrath, Michele C. Connelly, R. Kiplin Guy, Robert C. Reynolds

**Affiliations:** 1 *Drug Discovery Division, Southern Research Institute, 2000 Ninth Avenue South, Birmingham, AL 35205, USA*; 2 Drug Discovery Unit, College of Life Sciences, University of Dundee, Dundee DD1 5EH, United Kingdom; 3 Department of Chemical Biology & Therapeutics, St Jude Children's Research Hospital, 262 Danny Thomas Place, Mailstop 1000, Memphis, TN 38105-3678, USA; 4 The University of Kentucky College of Pharmacy, 214H BioPharm Complex, Lexington, KY 40536-0596, USA; 5 Division of Hematology and Oncology, The University of Alabama at Birmingham, Birmingham, Alabama 35294, USA

**Keywords:** NSAIDs, Sulindac, COX-independent, Reverse amide, Sulfonamide, Cancer, Anticancer

## Abstract

**Background:**

Sulindac belongs to the chemically diverse family of Non-Steroidal Anti-Inflammatory Drugs (NSAIDs) that effectively prevent adenomatous colorectal polyps and colon cancer, especially in patients with familial adenomatous polyposis. Sulindac sulfide amide (SSA), an amide analog of sulindac sulfide, shows insignificant COX-related activity and toxicity while enhancing anticancer activity *in vitro* and demonstrating *in vivo* xenograft activity.

**Objective:**

Develop structure-activity relationships in the sulindac amine series and identify analogs with promising anticancer activities.

**Method:**

A series of sulindac amine analogs were designed and synthesized and then further modified in a “libraries from libraries” approach to produce amide, sulfonamide and N,N-disubstituted sulindac amine sub-libraries. All analogs were screened against three cancer cell lines (prostate, colon and breast).

**Results:**

Several active compounds were identified *via* *in vitro* cancer cell line screening with the most potent compound (**26**) in the nanomolar range.

**Conclusion:**

Compound **26** and analogs showing the most potent inhibitory activity may be considered for further design and optimization efforts as anticancer hit scaffolds.

## 1 INTRODUCTION

Non-Steroidal Anti-Inflammatory Drugs (NSAIDs) are a structurally diverse group of agents that are used to treat chronic inflammatory diseases such as rheumatoid arthritis as well as acute symptoms of inflammation. Certain NSAIDs are also effective antipyretics and analgesics. The pharmacological basis for their anti-inflammatory activity involves the inhibition of COX isozymes (COX-1 and COX-2) and blockage of arachidonic acid conversion to prostaglandin H_2_, a precursor for the synthesis of prostaglandins, prostacyclins, and thromboxanes [1]. COX-1 is a constitutively expressed enzyme responsible for the regulation of prostaglandin biosynthesis in normal tissues and serves an important role in gastric cytoprotection, renal homeostasis, and platelet activation, while COX-2 is selectively and acutely expressed by inflammatory cells and certain cancers. Evidence from experimental and epidemiological studies, and clinical trials suggests that the regular use of NSAIDs can reduce the incidence and mortality of colorectal cancer by as much as 50% [2, 3]. The most compelling evidence for the role of NSAIDs in the prevention of colorectal tumors comes from clinical studies in patients with familial adenomatous polyposis (FAP) [4, 5]. However, chronic COX inhibition is associated with gastrointestinal, renal, and cardiovascular side effects, limiting the use of NSAIDs and COX-2 selective inhibitors for cancer chemoprevention [6-10]. NSAIDs are believed to display their anticancer effects through inhibition of COX-2, as this isozyme is thought to play a role in carcinogenesis and is often over expressed in human premalignant and malignant tissues [11-13]. Other published work suggests that NSAIDs may have COX/COX-2 independent effects. For example, cells lacking COX-1 and/or COX-2 show comparable sensitivity to NSAID-induced apoptosis, and, furthermore, NSAIDs that do not significantly inhibit COX-2 also induce apoptosis and inhibit carcinogenesis [14-17]. Accumulating evidence for COX-independent mechanisms underlying certain anticancer activities of the NSAIDs have been recently reviewed [18, 19]. A number of chemically modified NSAIDs has been also developed aiming to improve the efficacy and safety of conventional NSAIDs [20].

Sulindac belongs to the chemically diverse group of Non-Steroidal Anti-Inflammatory Drugs (NSAIDs) that effectively prevent adenomatous colorectal polyps and colon cancer, especially in patients with familial adenomatous polyposis [21-25]. Exisulind (sulindac sulfone), an oxidative metabolite of sulindac that lacks cyclooxygenase (COX) inhibitory activity, has also been shown to decrease polyp size and number in familial adenomatous polyposis patients, inhibit chemical carcinogenesis in rodents, and inhibit growth and induce apoptosis in a variety of human cancer cell lines [26-29]. As reported previously, Sulindac Sulfide Amide (**SSA**, Fig. (**1**), obtained from sulindac sulfide (**1**) and N,N-dimethylethylenediamine replacing the carboxylate moiety in sulindac, shows severely attenuated COX inhibition while demonstrating excellent anticancer activity *in vitro* compared to the parent compound sulindac sulfide as well as having *in vivo* murine xenograft activity [30]. Hence, we have synthesized additional sulindac libraries in order to further probe the anticancer activity of this promising class of NSAIDs.

Clearly, numerous alternative targets other than the COX isozymes have specifically been implicated in the activity of sulindac including platelet activating factor (PAF), retinoid receptors (RXRα and RXRα-dependent AKT signaling), cyclic-GMP phosphodiesterase, peroxisome proliferator-activated receptor (PPARγ), 5-lipoxygenase, Smad2/3, Wnt/ß-catenin, NF-κB, Ras/Raf/P53 pathways, and specificity protein transcription factors (Sp) among others [reviewed in 18, 31]. In fact, at a recent meeting to discuss alternative targets for the NSAIDs, it was stated “. . . at the very least, the studies finding non-COX-2-related effects of NSAIDS are identifying potential new targets for drugs that can be used to prevent or treat cancer” [32]

**Fig. (1):**
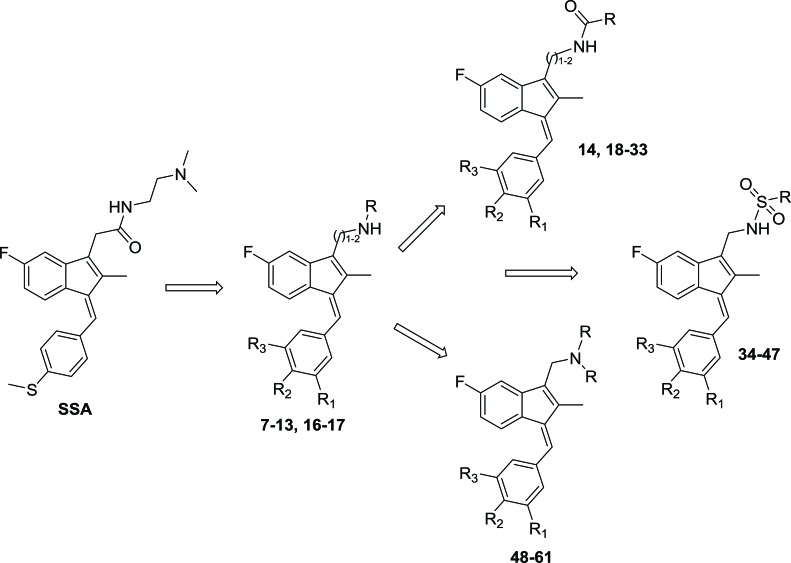
Overview of structural modifications related to SSA.

Hence, it is our hypothesis that diversity libraries built around the classical, “privileged” NSAID scaffolds will show numerous and interesting biological activities that can be used to study the chemical biology of this class with a goal of exploring interesting potential targets beyond the cyclooxygenases for new drug discovery. The fact that both Exisuland and our lead SSA showed severely attenuated COX inhibition was a strong basis for preparing similar analogs that showed abrogated COX inhibition allowing us to potentially better explore “off target” activities of the sulindac scaffold. The lack of COX-2 inhibition of SSA supports our modelling-based hypothesis that replacement of the carboxylate moiety of sulindac with basic substituents may attenuate or abolish COX-2 binding of analogs derived from sulindac (described below). Further substitution vectors for designing out COX-2 activity are predicted in the benzylidene moiety of sulindac, particularly in 3-, 4-, 5-positions of benzene. We designed a variety of series that explore these opportunities to remove COX inhibition while potentially expanding into non-COX active anticancer space. In the present manuscript, we describe the synthesis and anticancer activity of a series of sulindac ethane amines and sulindac methane amines (**7-13, 16-17**) as shown in Fig. (**1**). These compounds were further modified to amide (**14, 18-33**), sulfonamide (**34-47**) and N,N-disubstituted sulindac amine (**48-61**) libraries in order to further probe anticancer activity of this class (Fig. **1**).

## 2. Materials and Methods

### 2.1 Chemistry

The general synthetic route used to prepare the target compounds **7-12** is outlined in Scheme (**1**). Synthesis started with the conversion of sulindac, sulindac sulfide or sulindac sulfone (**1-3**) to the methyl esters, which were converted to the corresponding aldehydes (**4-6**) by treating with DIBALH. Finally, reductive amination of the aldehydes with various substituted amines afforded compounds **7-12** in moderate to good yields [33].

Sulindac sulfide ethane amine **13** was prepared from sulindac sulfide (**1**) following the four-step sequence shown in Scheme (**2**). The sequence involved the reduction of the acid to the corresponding alcohol, which was then converted to an iodide by treating with tetrabutylammonium iodide, pyridine and trifluoromethanesulfonic anhydride [34]. The iodide was then converted to the corresponding azide using sodium azide followed by a Staudinger type reduction [35] to give sulindac sulfide ethane amine **13**. Amine **13** was further transformed to amide **14** using standard peptide coupling conditions.

Methane amine analogs (**16-17**) of different sulindac derivatives were synthesized from their corresponding acids (**1, 15**) by treating with oxalyl chloride followed by trimethylsilyl azide and acetic acid [34]. These amines were further modified in three ways: 1) conversion to amides by reacting with various acids (**18-33**); 2) conversion to sulfonamides by reacting with sulfonyl chlorides and pyridine (**34-47**) and; 3) reductive amination of **16** or **17** with various aldehydes and Na(OAc)_3_BH to afford bis amino substituted sulindac methane amines (**48-61**) Scheme (**3**) [33].

## 3 RESULTS AND DISCUSSION

### 3.1 Screening Results

Using quantitative high-throughput screens (qHTS) all synthesized compounds were screened against three cancer cell lines: HT29 colorectal carcinoma, PC3 prostate and MDA-MB-231 breast cancer cell lines according to reported methods – see Supplementary Material, Appendix A. Screening results are summarized in (Tables **1**-**6**).

Table (**1**) lists the anticancer activity of compounds **7-12** in colon, prostate and breast cancer cell lines. Compound **10**, the amine analog of SSA, was found to be active, but slightly less potent than SSA. Compound **9** with a 4-methylthiobenzylidene ring at the C-1 position and 1-pipyridinylethylaminoethyl group at the C-3 position had activity very similar to **10**. Among benzylaminoethyl analogs of sulindac sulfide, sulfoxide and sulfone (**7**, **11** and **12**), the sulindac sulfone analog **12** displayed significant anticancer activity. The furan-2-ylmethylaminoethyl analog of sulindac sulfide (**8**) was active against HT29, PC3 and MDA-MB-231 cells, but was slightly less potent than SSA.

Sulindac sulfide ethane amine **13** Table (**2**) was prepared to study the role of substituents at the amino ethane group. Compound **13** was modestly less active than its 1-pipyridinylethyl and N,N-dimethylaminoethyl counterparts **9** and **10**, but more active than its benzyl derivative **7** (Table **1**). No significant differences were observed in the case of **13** and its furan-2-ylmethyl analog **8**. These results demonstrate that appropriate amino ethane analogs show significant activity relative to sulindac and its metabolites as well as other analogs. Conversion of **13** to its reverse amide analog **14** slightly reduced potency by two fold, and the reverse amide was also modestly less potent than reduced analog **8**.

Two analogs **16** and **17** Table (**3**) were prepared to study the effect of substituting an amino methyl linker for the amino ethyl linker in **13**. Both analogs were less active than **13** against all three cancer cell lines, while their potencies were comparable.

Compounds **16** and **17** were further modified to reverse amide analogs **18-33** Table (**4**), sulfonamide derivatives **34-47** Table (**5**) and disubstituted amino derivatives **48-61** Table (**6**) to explore the role of different functional groups in anticancer activity. Compounds **18-33**, which contain an amide moiety at the C-3 position, displayed significant anticancer activity in all the three cell lines except their furan-2-yl analogs **19** and **27**. Importantly, 3,4,5-trimethoxybenzylidene-3-indenylmethyl-2-phenylacetamide (**26**) was the most effective and potent inhibitor of cancer cell growth among all the compounds tested, with CC_50_ of <98.0 nM in colon and breast cancer cells and 520.0 nM in prostate cancer cells. Among all the sulfonamide derivatives **34-47**, 4-thiomethylbenzylidine analogs **34-40** were more active than their 3,4,5-trimethoxybenzylidine analogs **41-47**. Conversion of methaneamine linker to a series of disubstituted methaneamine compounds reduced potency, except for the pyridyl analogs **49** and **56**.

### 3.2 Cytotoxicity Evaluations and Screening Against a Panel of Additional Cancer Cell Lines

Most analogs in the presented series are lipophilic while also containing a positively charged amine. Lipophilic basic amines can accumulate in cell membranes if their lipophilic atom groups form favorable non-polar contacts with lipid chains while their charged amine is interacting with fatty acid head groups. Such association with lipids can lead to cytotoxicity through membrane destabilization. Therefore, we evaluated a number of representative compounds against BJ cells, a normal human foreskin fibroblast cell line, where cytotoxicity was determined at 10 µM drug concentration, as described previously [36]. Results of cytotoxicity evaluations for selected compounds are shown in Table **S-1** (Supplementary materials: Appendix A). BJ cytotoxicity evaluation suggests that these compounds are not cytotoxic. In particular, compound **26** possessing the highest potency against all three cancer cell lines shows EC_50_ > 22.73 µM in the BJ cytotoxicity assay. Further, Table **S1** includes screening results against a panel of additional cancer cell lines performed at St. Jude Children’s Research Hospital for selected compounds (as described in Supplementary materials: Appendix A). Compound **26** stands out as having inhibitory activity against five acute lymphoblastic leukemia cell lines used on this panel (Table **S1**), and thus this compound may be considered a viable lead for further development. Compounds **20**, **23**, **24**, **26**, **32** and **56** were active against multiple leukemia cell lines showing a wide range of efficacy while also inhibiting the three (HT29, PC3, MDA-MB-231) cancer cell lines with CC_50_ < 10 µM potency.

### 3.3 Sulindac Amine Derivatives *Versus* Sulindac Modelled into COX-2

The binding mode of sulindac, a known COX-2 inhibitor was predicted based on the X-ray structure of COX-2 (PDB code 4COX) using Induced Fit docking as implemented in Schrödinger software Fig. (**2**). The carboxylate group of sulindac forms a hydrogen bond with Y355 and highly favorable, salt bridging/hydrogen bonding interactions with R120, while the conformation of the R120 side chain is restrained by a salt bridge to E524. These favorable contacts are also present in the X-ray structure of COX-2 (PDB code 4COX) through the carboxylate group of co-crystallized indomethacin. Docking places the carboxylate of sulindac in overlapping position with that of indomethacin co-crystallized in COX-2.

The carboxylate functionality of sulindac was replaced by various basic amine containing groups in the presented amine derivative series, none of which are suitable for forming favorable polar interactions with Arg120. In addition to the loss of salt bridging and hydrogen bonding interactions, basic amine groups close to the guanidinium moiety of R120 would not be favorable. This is illustrated by superimposing a representative analog (**10**) onto sulindac in Fig. (**2**). Compound **10** could not be docked favorably into COX-2, even while treating residues in the binding site region as flexible. Compound **10** has a very similar structure to SSA (sulindac sulfide amide). The difference between their structures is that an amide group in SSA is replaced by a more basic methylene-amine group in compound **10** Fig. (**1**), Scheme (**1**). Superposition of compound **10** onto sulindac places this group in approximately overlapping space with the carboxylate of sulindac and near R120 Fig. (**2**). Interestingly, SSA inhibits human colon tumor cell lines while showing no activity against COX-1 and COX-2 [30]. The lack of COX-2 activity of SSA supports our analogous, model based prediction that compound **10** and its analogs containing basic amine substituents are unlikely to inhibit COX-2. Similar reasoning was incorporated in SSA analog designs previously as in our approach to design out COX-2 inhibitory activity through replacement of the carboxylate of sulindac with a variety of amine containing substituents [15].

The docked pose of sulindac suggests further vectors to explore for the design of new analogs inactive against COX-2. In the docked pose of sulindac the benzylidene moiety is accommodated in a deep-lying pocket, surrounded by largely non-polar residues as shown in Fig. (**2**): F381, L384, W387, F518, M522, V523, L352, as well as (residues not shown for clarity) G526, A527, Y385. Tight packing of residues around the benzene ring especially in the region of C3, C5 include the side chains of Y385, W387 and backbone atoms of M522, V523, which restrict pocket volume available for 3-, 5- substituents. The 4-methylsulfinyl substituent is fitted tightly in a small cavity forming a hydrogen bonding interaction with W387. Limited cavity space in this region suggests that 3-, 4-, 5-substitutions of the benzene ring may lead to steric hindrance, interfering with the binding of sulindac analogs to COX-2. Therefore benzene substitutions have been also explored in the presented analog series.

### 3.4 Computed Physicochemical Properties

Favorable permeability and metabolic stability are key properties relevant to oral bioavailability that should be considered early on during lead optimization. In order to assess physicochemical properties of our most potent series (**7**-**33**) we correlated logD and molecular weight (MW) of the compounds and selected the analogs that map to an optimal ‘Golden Triangle’ region as shown in Fig. (**3**), as proposed based on results of previous studies [37, 38]. For example, Johnson and co-workers [37] used *in vitro* permeability data available in Caco-2 cells for 16,227 compounds and *in vitro* metabolic clearance data derived from human liver microsome (HLM) stability for 47,018 compounds for a correlation analysis with computed physicochemical properties [37]. Among physicochemical properties, logD was found to positively correlate with permeability, except at high logD values. Metabolic clearance was negatively correlated with logD and molecular weight (MW). Permeability and HLM stability data have been combined in order to deduce the optimal ranges for logD and MW which describe compounds that possess both, good permeability and metabolic stability properties. The optimal ranges map approximately to a triangular area (‘Golden Triangle’) in a plot of MW *versus* logD (with a baseline at MW 200 between logD -2 to 5 and apex at MW 450 between logD 1 – 2). Similar results have been obtained based on the logD and Caco-2 permeability data analysis of 9,571 structurally diverse compounds [38]. Therefore, we chose to correlate MW and logD for the compounds in our series and identify those that map to or near the optimal ranges. Fig. (**3**) illustrates several active analogs that map to the “goldilocks area” (**13**, **16**, **17**) or near the borderline of this optimal zone (**21**, **29**, **30**), which may be associated with desirable *in vivo* permeability and metabolic clearance properties based on the mentioned published findings. Table **S2** (Supplementary Materials: **Appendix A**) lists computed physicochemical properties for the series **7**-**33**.

## CONCLUSION

In summary, we prepared a series of sulindac analogs with significant anticancer activity in HT29, PC3 and MDA-MB-231 cells, exploring synthetic vectors suggested by our modelling results. A number of new compounds have been identified that maintain potent inhibitory activity against the three cancer cell lines and additionally show improved computed physicochemical properties. For example, compounds **13**, **16**, **17**, **21**, **29, 30** possess logD and MW values that map to the Golden Triangle region of property space that has been associated with better *in vivo* permeability and improved metabolic stability properties. Compound **26** shows significant anticancer activity in all three cell lines and was active against five acute lymphoblastic leukemia cell lines as well. In comparison with our original lead SSA, **26** displayed increased anticancer activity by 6-7-fold in colon and prostate cancer cells and by 27-fold in breast cancer cells. Although **26** possesses higher lipophilicity (logD 5.13) than desirable, this compound may serve as a starting scaffold for further design and optimization efforts. Based on the presented results, several compounds are candidates for mechanistic studies and *in vivo* evaluations, and these advanced assays will be part of separate studies. This new library of sulindac/SSA analogs with the associated cancer cell line growth inhibition data will hopefully provide interested researchers with a basis for advancing s or related active analogs into advanced *in vitro* and in vivo studies that will expand our knowledge of the anticancer properties and targets of the NSAIDs.

## SUPPLEMENTARY MATERIALS

### Appendix A

Screening against HT29 colorectal carcinoma, PC3 prostate and MDA-MB-231 breast cancer cell lines are described. Results of additional cancer cell line screens and cytotoxicity data is summarized in Table **S1**. Computed physicochemical properties of compounds **7**-**33** are listed in Table **S2**.

### Appendix B

General experimental methods, synthetic procedures and analytical data are provided.

## Figures and Tables

**Fig. (2):**
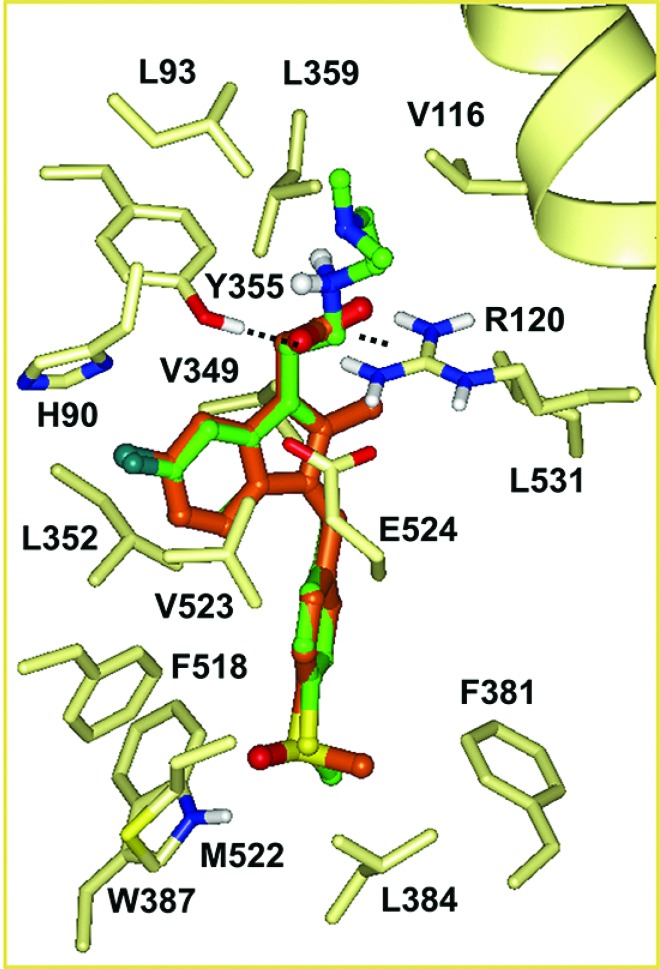
Sulindac (orange carbons) docked into the COX-2 active site and the superimposed compound **10** (green colored carbons).

**Fig. (3):**
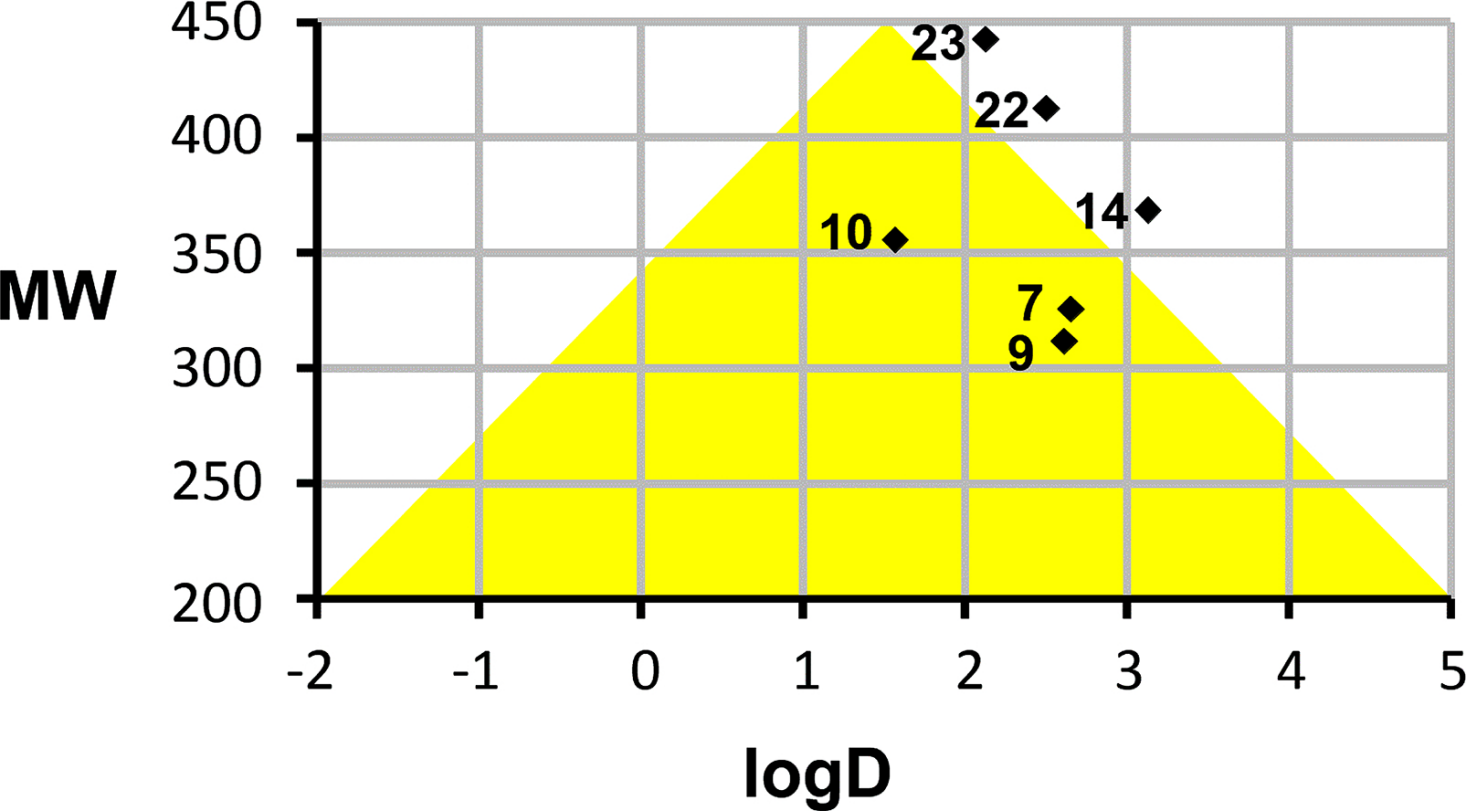
Sulindac (orange carbons) docked into the COX-2 active site and the superimposed compound **10** (green colored carbons).

**Scheme (1):**
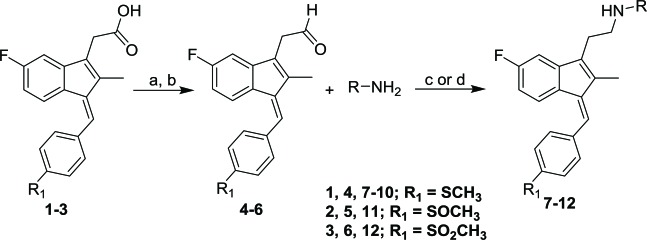
Synthetic pathways to analogs **7-12**. Reagents and conditions: (a) MeOH, SOCl_2_ (b) DIBAL-H, Toluene (c) NaBH_4_, MeOH, rt (d) Na(OAc)_3_BH, 1,2-DCE, rt.

**Scheme (2):**
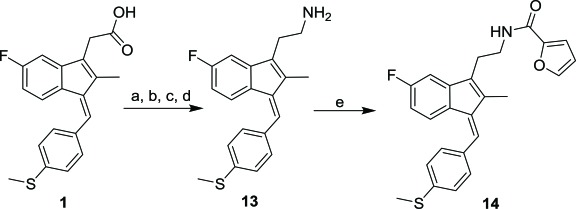
Synthetic pathways to analogs **13-14**. Reagents and conditions: (a) BH_3_/THF, 0 ^o^C-rt (b) TBAI, Pyridine, CH_2_Cl_2_, Tf_2_O, -78 ^o^C-rt (c) NaN_3_, MeCN, reflux (d) PPh_3_, rt (e) 2-Furoic acid, HBTU, Et_3_N, MeCN, rt.

**Scheme (3):**
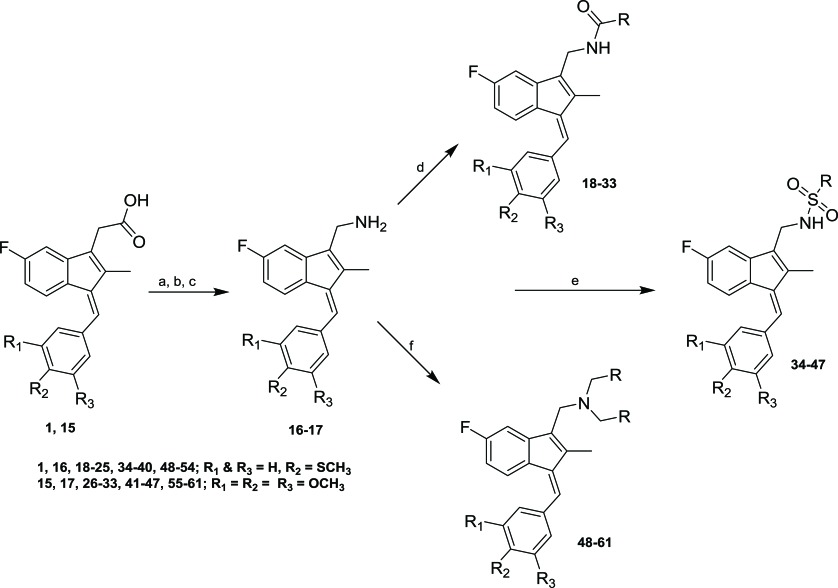
Synthetic pathways to analogs **14-59**. Reagents and conditions: (a) Oxalyl chloride, DMF, CH_2_Cl_2_, rt (b) trimethylsilylazide, CCl_4_, rt-50 ^o^C (c) AcOH/HCl (d) RCO_2_H, HATU, DIEA, MeCN (e) RSO_2_Cl, NMI, Pyridine (f) RCHO, Na(OAc)_3_BH, 1,2-DCE, rt

**Table 1:** Screening data for compounds **7-12**.

**Cmpd**	**R & R_1_**	**CC_50_ (*µ*M)**		
		**HT29**	**PC3**	**MDA-MB-231**
**SSA**		0.65±0.03	3.12±0.15	2.67±0.08
**7**	R_1_ = SCH_3_, R = benzyl	21.83±2.29	>50.00	46.51±1.92
**8**	R_1_ = SCH_3_, R = furan-2-ylmethyl	4.27±0.33	9.94±0.77	7.77±0.54
**9**	R_1_ = SCH_3_, R = 1-pipyridinylethyl	1.81±0.06	4.78±0.20	3.73±0.21
**10**	R_1_ = SCH_3_, R = CH_2_CH_2_N(CH_3_)_2_	1.97±0.18	4.38±0.25	3.42±1.50
**11**	R_1_ = SOCH_3_, R = benzyl	15.14±1.42	41.46±5.00	48.57±7.85
**12**	R_1_ = SO_2_CH_3_, R = benzyl	3.39±0.28	7.02±0.48	4.52±0.77

**Table 2:** Screening data for compounds **13-14**.

**Cmpd**	**CC_50_ (*µ*M)**		
	**HT29**	**PC3**	**MDA-MB-231**
**13**	4.05±0.16	7.46±0.46	7.20±0.29
**14**	9.24±1.18	8.92±0.83	16.78±3.20

**Table 3:** Screening data for compounds **16-17**.

**Cmpd**	**R_1_, R_2_ & R_3_**	**CC_50_ (*µ*M)**		
		**HT29**	**PC3**	**MDA-MB-231**
**16**	R_1_ & R_3_ = H, R_2_ = SCH_3_	15.99±6.08	33.60±6.59	19.33±2.12
**17**	R_1_, R_2_ & R_3_ = OCH_3_	14.64±2.42	29.73±4.49	15.35±4.44

**Table 4:** Screening data for compounds **18-33**.

**Cmpd**	**R, R_1_, R_2_ & R_3_**	**CC_50_ (*µ*M)**		
		**HT29**	**PC3**	**MDA-MB-231**
**18**	R_1_ & R_3_ = H, R_2_ = SCH_3_, R = benzyl	3.56±1.25	18.86±9.71	4.06±1.97
**19**	R_1_ & R_3_ = H, R_2_ = SCH_3_, R = furan-2-yl	33.25±3.43	39.79±6.81	41.16±7.60
**20**	R_1_ & R_3_ = H, R_2_ = SCH_3_, R = > 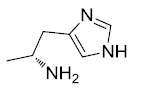	3.64±0.21	4.81±0.99	7.24±0.56
**21**	R_1_ & R_3_ = H, R_2_ = SCH_3_, R = aminomethyl	5.49±0.21	7.27±0.31	7.30±0.62
**22**	R_1_ & R_3_ = H, R_2_ = SCH_3_, R = 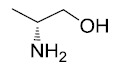	5.98±0.24	6.03±0.41	7.43±0.87
**23**	R_1_ & R_3_ = H, R_2_ = SCH_3_, R = 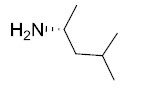	4.39±0.29	6.21±0.49	6.70±0.71
**24**	R_1_ & R_3_ = H, R_2_ = SCH_3_, R = 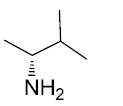	4.23±0.16	5.16±0.50	6.81±0.63
**25**	R_1_ & R_3_ = H, R_2_ = SCH_3_, R = 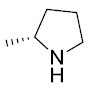	3.15±0.17	6.66±0.42	4.35±0.19
**26**	R_1_, R_2_ & R_3_ = OCH_3_, R = benzyl	<0.098±0	0.52±0	<0.098±0.04
**27**	R_1_, R_2_ & R_3_ = OCH_3_, R = furan-2-yl	>50.00	>50.00	>50.00
**28**	R_1_, R_2_ & R_3_ = OCH_3_, R = methyl	6.44±1.40	23.26±2.61	5.77±1.55
**29**	R_1_, R_2_ & R_3_ = OCH_3_, R = aminomethyl	8.51±1.70	17.92±2.08	9.92±2.13
**30**	R_1_, R_2_ & R_3_ = OCH_3_, R = 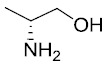	11.05±1.35	26.53±6.01	11.94±2.27
**31**	R_1_, R_2_ & R_3_ = OCH_3_, R = 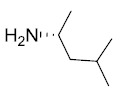	5.21±0.39	11.95±1.47	8.87±1.38
**32**	R_1_, R_2_ & R_3_ = OCH_3_, R = 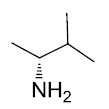	4.87±0.11	8.11±0.41	7.30±0.73
**33**	R_1_, R_2_ & R_3_ = OCH_3_, R = 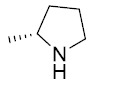	5.67±1.67	11.94±1.56	8.20±0.99

**Table 5:** Screening data for compounds **34-47**.

**Cmpd**	**R, R_1_, R_2_ & R_3_**	**CC_50_ (*µ*M)**		
		**HT29**	**PC3**	**MDA-MB-231**
**34**	R_1_ & R_3_ = H, R_2_ = SCH_3_, R = methyl	7.43±0.48	20.18±3.20	9.06±2.26
**35**	R_1_ & R_3_ = H, R_2_ = SCH_3_, R = ethyl	8.33±1.64	22.59±3.25	11.87±2.61
**36**	R_1_ & R_3_ = H, R_2_ = SCH_3_, R = phenyl	9.94±0.83	39.58±14.8	17.72±2.10
**37**	R_1_ & R_3_ = H, R_2_ = SCH_3_, R = *p*-tolyl	8.08±0.60	>50.00	20.53±5.18
**38**	R_1_ & R_3_ = H, R_2_ = SCH_3_, R = 1-naphthyl	17.03±3.73	>50.00	38.90±10.32
**39**	R_1_ & R_3_ = H, R_2_ = SCH_3_, R = 2-naphthyl	25.64±5.91	>50.00	12.92±2.39
**40**	R_1_ & R_3_ = H, R_2_ = SCH_3_, R = 5-N,N-Dimethylamino-1-naphthyl	38.41±10.12	>50.00	>50.00
**41**	R_1_, R_2_ & R_3_ = OCH_3_, R = methyl	20.02±4.86	18.85±2.47	12.91±1.79
**42**	R_1_, R_2_ & R_3_ = OCH_3_, R = ethyl	15.56±5.56	15.13±1.84	6.58±1.39
**43**	R_1_, R_2_ & R_3_ = OCH_3_, R = phenyl	43.77±23.02	40.88±16.80	16.28±3.96
**44**	R_1_, R_2_ & R_3_ = OCH_3_, R = *p*-tolyl	>50.00	>50.00	44.48±25.65
**45**	R_1_, R_2_ & R_3_ = OCH_3_, R = 1-naphthyl	>50.00	>50.00	>50.00
**46**	R_1_, R_2_ & R_3_ = OCH_3_, R = 2-naphthyl	>50.00	>50.00	>50.00
**47**	R_1_, R_2_ & R_3_ = OCH_3_, R = 5-N,N-Dimethylamino-1-naphthyl	>50.00	>50.00	>50.00

**Table 6:** Screening data for compounds **48-61**.

**Cmpd**	**R, R_1_, R_2_ & R_3_**	**CC_50_ (*µ*M)**		
		**HT29**	**PC3**	**MDA-MB-231**
**48**	R_1_ & R_3_ = H, R_2_ = SCH_3_, R = phenyl	>50.00	>50.00	>50.00
**49**	R_1_ & R_3_ = H, R_2_ = SCH_3_, R = 4-pyridyl	10.76±1.84	12.73±1.44	9.50±2.14
**50**	R_1_ & R_3_ = H, R_2_ = SCH_3_, R = 4-Fluorophenyl	>50.00	>50.00	>50.00
**51**	R_1_ & R_3_ = H, R_2_ = SCH_3_, R = 4-N,N-Dimethylaminophenyl	>50.00	>50.00	>50.00
**52**	R_1_ & R_3_ = H, R_2_ = SCH_3_, R = 2-naphthyl	>50.00	>50.00	>50.00
**53**	R_1_ & R_3_ = H, R_2_ = SCH_3_, R = benzyl	>50.00	>50.00	>50.00
**54**	R_1_ & R_3_ = H, R_2_ = SCH_3_, R = furan-2-yl	>50.00	>50.00	48.76±20.34
**55**	R_1_, R_2_ & R_3_ = OCH_3_, R = phenyl	>50.00	>50.00	>50.00
**56**	R_1_, R_2_ & R_3_ = OCH_3_, R = 4-pyridyl	9.83±1.54	9.09±0.87	8.21±0.90
**57**	R_1_, R_2_ & R_3_ = OCH_3_, R = 4-fluorophenyl	>50.00	>50.00	>50.00
**58**	R_1_, R_2_ & R_3_ = OCH_3_, R = 2-naphthyl	>50.00	>50.00	>50.00
**59**	R_1_, R_2_ & R_3_ = OCH_3_, R = 4-methoxyphenyl	>50.00	>50.00	>50.00
**60**	R_1_, R_2_ & R_3_ = OCH_3_, R = benzyl	>50.00	>50.00	>50.00
**61**	R_1_, R_2_ & R_3_ = OCH_3_, R = furan-2-yl	>50.00	>50.00	>50.00
