# Supplementary material for: Amine Containing Analogs of Sulindac for Cancer Prevention
Source: Open Med Chem J. 2018 Jan 31;12:1–12. doi: 10.2174/1874104501812010001 (PMC5817852; doi:10.2174/1874104501812010001)
Supplement: Supplementary file 1 [file TOMCJ-12-1_SD1.pdf]

Send Orders for Reprints to reprints@benthamscience.ae

The Open Medicinal Chemistry Journal, 2018, *12*, i-xiv

BENTHAM OPEN

CrossMark

The Open Medicinal Chemistry Journal

Supplementary Material

Content list available at: www.benthamopen.com/TOMCJ/

DOI: 10.2174/1874104501812010001

Amine Containing Analogs of Sulindac for Cancer Prevention

Bini Mathew[1], Judith V. Hobrath[2], Michele C. Connelly[3], R. Kiplin Guy and Robert C. Reynolds[5,*]

[1]*Drug Discovery Division, Southern Research Institute, 2000 Ninth Avenue South, Birmingham, AL 35205, USA*
[2]*Drug Discovery Unit, College of Life Sciences, University of Dundee, Dundee DD1 5EH, United Kingdom*
[3]*Department of Chemical Biology & Therapeutics, St Jude Children's Research Hospital, 262 Danny Thomas Place, Mailstop 1000, Memphis, TN38105-3678, USA*
[4]*The University of Kentucky College of Pharmacy, 214H BioPharm Complex, Lexington, KY40536-0596, USA*
[5]*Division of Hematology and Oncology, The University of Alabama at Birmingham, Birmingham, Alabama35294, USA*

Received: November 1, 2017 Revised: January 17, 2018 Accepted: January 21, 2018

SUPPLEMENTARY MATERIALS: APPENDIX A

1. GENERAL EXPERIMENTAL METHODS

All target sulindac analogs were screened using quantitative high-throughput screen (qHTS) against prostate, colon and breast cancer cell lines. Liquid handling was performed on a Biomek FX with a 384-multichannel head. In 384 well plates, compounds were arrayed in columns 3-22 leaving 32 wells for positive and negative controls. All compounds were diluted together in a plate to plate transfer. Cells were then added to assay plates containing diluted compound using a Matrix/Thermo wellmate. Cells were incubated with compound for three cell doublings. Due to differences in growth rates between cell lines, the incubation period for PC3 and HT-29 cells was 72 hours, but was increased to 96 hours for MDA-MB-231 cells. Plates were incubated for the appropriate time 72 or 96 hours and cell viability determined using Cell Titer Glo (Promega). The dose response format employed a cross-plate method rather than an in-plate method, allowing for more efficient compound dilution and addition to assay plates. Two-fold dilutions of the compound mother plate were aliquoted to a series of 384-well plates using a stacked plate (or cross-plate) format. Object manager was used to create the assay plates by replicating the compound mother plate and assigning concentration values to the assay plates. Luminescence values were read on the envision plate reader for each of the assay plates. The entire experiment of assay plates was set up in a single day with a complete read of all plates occurring at 72 hours and or at 96 hours as required for the cell lines. Data were imported and analyzed within 24 hours of the endpoint read. From set up to final report, all data points were generated and reported within one week. Therefore, only a single passage was required for each cell line, eliminating potential variation due to passage and cell count. For data analysis Activity Base software (IDBS) was used. The data were imported into the database and calculated using an ActivityBase XE template where the Virtual Plate functionality was employed to maintain the link between the assay plates and the compound mother plate from which they were created. For each plate the median, standard deviations, CVs and Z values were calculated for the control wells. These values were used to assure quality and consistency across all test plates and to normalize percent cell viability for each well. XLFit and MathIQ were used within the ActivityBase XE template to plot the dose response curves and calculate CC_{50} values. The CC_{50}s were calculated by plotting the cell viability relative to the mean of the cell control at each of the tested compound concentration. Compounds that caused cell viability < 80% were considered active. Values were calculated only for active compounds using a 4-parameter Levenburg-Marquardt algorithm (XLFit #205), with the maximum and

minimum locked at 0 and 100 respectively. Data and graphical results were then reported and compared across the three cell lines.

2. SCREENING RESULTS AGAINST A PANEL OF CANCER CELL LINES AND CYTOTOXICITY DATA

A panel of additional cancer cell lines were used for screening selected compounds. Results are summarized in Table (**1**). The following cell lines were included in the panel: a. CPC300 cells derived from a mouse model of choroid plexus carcinoma (CPC), where the mouse model has the genetic background: Trp53LoxP-RBLoxP-PtenLoxP; b. NALM16 cell line established from the peripheral blood of a 12 year old girl with acute lymphoblastic leukemia (ALL) at relapse in 1977 carrying a near haploid karyotype; c. Burkitt's leukaemia/lymphoma, with FAB L3 (RAJI cells); d. Acute T cell leukemia Jurkat e6-1 cells(JURKT); e. Precursor B-cell ALL patient-derived cell line expressing only wild-type MLL and wild-type AF4 (REH cells); f. Cell line (697) established from bone marrow cells obtained from children with ALL in relapse. Compounds were counter screened for cytotoxicity in a BJ cell assay, a normal human foreskin fibroblast cell line (BJ). Sulindac sulfide amide inactivity against the cell lines in this panel.

Table 1. Cancer cell line screening and cytotoxicity data. Confidence intervals are shown in parentheses; NA: not active; ND: undetermined due to questionable curve fit.

Cmpds		EC_{50} (μM)					
	CPC300	NALM16	RAJI	JURKT	REH	697	BJ
**SSA**	NA	NA	NA	NA	NA	NA	>7.57
**7**	NA	NA	NA	NA	NA	NA	>7.57
**8**	NA	NA	NA	NA	NA	NA	>7.57
**9**	NA	NA	NA	NA	ND	NA	>7.57
**13**	NA	NA	NA	NA	NA	NA	>7.57
**14**	NA	NA	NA	NA	NA	NA	>7.57
**20**	NA	NA	45 (43-47)	NA	14 (13-14)	NA	14.63 (13.45-15.92)
**21**	NA	NA	NA	NA	NA	NA	>22.73
**22**	NA	NA	NA	NA	NA	NA	>22.73
**23**	NA	30 (29-31)	NA	NA	16 (16-16)	NA	16.39 (3.80-70.79)
**24**	NA	NA	39(38-42)	15 (15-15)	14 (13-14)	16 (16-16)	16.74 (3.57-78.47)
**25**	NA	NA	NA	NA	150 (120-180)	NA	>22.73
**26**	NA	8.1 (8-8.3)	8.3 (8.1-9.2)	11 (9.5-12)	19 (18-19)	15 (14-15)	>22.73
**29**	NA	230 (130-230)	NA	NA	NA	NA	>22.73
**30**	NA	NA	NA	NA	53 (45-63)	NA	>22.73
**31**	NA	NA	NA	NA	16 (15-16)	NA	>22.73
**32**	NA	NA	110 (97-150)	NA	21 (21-21)	NA	>22.73
**33**	NA	NA	NA	33 (33-47)	38 (33-44)	NA	>22.73
**34**	NA	ND	ND	NA	NA	NA	>22.73
**49**	NA	0.051 (0.033-0.089)	NA	NA	NA	NA	>22.73
**56**	NA	32 (31-34)	58 (48-76)	NA	32 (31-33)	NA	>22.73

3. COMPUTED PHYSICOCHEMICAL PROPERTIES

Table 2. Computed physicochemical properties for the series 7-33 using ACD (Percepta software). SSA: Sulindac sulfide amide, MW: molecular weight, TPSA: topological polar surface area; LogD computed at pH 7.4.

Cmpds	MW	LogD	LogP	TPSA
**SSA**	410.56	4.41	5.03	57.64
**7**	415.58	4.95	6.78	37.33
**8**	405.54	5.38	6.47	50.47
**9**	436.64	3.82	6.65	40.57
**10**	396.57	3.12	5.65	40.57
**11**	431.58	3.40	5.22	48.31
**12**	447.58	3.58	5.40	54.55
**13**	325.45	2.62	4.96	51.32

(Table 2) contd.....

Cmpds	MW	LogD	LogP	TPSA
**14**	419.52	5.68	5.68	67.54
**16**	311.42	2.66	4.65	51.32
**17**	355.41	1.57	3.55	53.71
**18**	429.56	5.91	5.91	54.4
**19**	405.49	5.44	5.44	67.54
**20**	448.57	2.93	3.28	109.10
**21**	368.48	3.13	3.32	80.42
**22**	398.50	3.06	3.29	100.65
**23**	424.59	4.51	5.14	80.42
**24**	410.56	4.22	4.85	80.42
**25**	408.54	3.87	4.84	66.43
**26**	473.55	5.13	5.13	56.79
**27**	449.48	4.60	4.60	69.93
**28**	397.45	3.57	3.57	56.79
**29**	412.47	2.50	2.69	82.81
**30**	442.49	2.13	2.36	103.04
**31**	468.57	3.36	3.98	82.81
**32**	454.55	3.03	3.65	82.81
**33**	452.53	2.69	3.66	68.82

4. DOCKING PROTOCOL

For docking the crystal structure of COX-2 (PDB code 4COX) was utilized following an initial refinement of the structure using the Protein Preparation Wizard implemented in the Schrodinger software. Induced Fit docking method (Schrodinger) was used, which combines Glide docking with Prime structural refinement tools and accounts for side chain flexibility near the ligand during docking. The center of the docking grid was defined as the centroid of the co-crystallized indomethacin in the crystal structure; the enclosing box size was set to 27 Å. Induced Fit parameters were at default values except docking was performed in extra precision mode.

SUPPLEMENTARY MATERIALS: APPENDIX B

1. GENERAL EXPERIMENTAL METHODS

Anhydrous solvents and reagents from Aldrich were used without further drying. Reactions were monitored by thin-layer chromatography (TLC) on precoated E. Merck silica gel (60F254) plates (0.25 mm) and visualized using UV light (254 nm). Flash chromatography was carried out on Fischer silica gel G 60 (230-400 mesh). Purification of certain compounds was carried out by utilizing a Teledyne Isco Combiflash® Rf automated chromatography machine. Melting points, determined with a OptiMelt Automated Melting Point System and, are uncorrected. The exact mass spectral data were obtained with an Agilent LC-MSTOF or with a Bruker BIOTOF II by electrospray ionization (ESI). ^{1}HNMR spectra were recorded on a Nicolet NT-300 NB spectrometer operating at 300.635 MHz or on Agilent/Varian MR-400 spectrometer operating at 399.930 MHz. Chemical shifts in $CDCl_3$ and Me_2SO-d_6 are expressed in parts per million downfield from tetramethylsilane (TMS) Chemical shifts (δ) listed for multiplets were measured from the approximate centers, and relative integrals of peak areas agreed with those expected for the assigned structures. Determination of % purity were obtained by HPLC using an Agilent 1100 LC equipped with a diode array UV detector and monitored at multiple wavelengths. ESI-MS spectra were recorded on a BioTof-2 time-of-flight mass spectrometer.

2. SYNTHETIC PROCEDURES

Method 1

To a suspension of acid (1 equivalent) in dry methanol at 0 ^{o}C was slowly added thionyl chloride (1.5 equivalents). The reaction mixture was stirred at room temperature for 3 h. Methanol was removed and the crude product was then washed with aqueous $NaHCO_3$ solution and extracted with $CHCl_3$ (2 x 25 mL). The combined organic extracts were dried over anhydrous Na_2SO_4 and the solvent was evaporated off under reduced pressure. Pure product was separated by crystallization.

Method 2

To a solution of ester (1 equivalent) in dry toluene at -70 ^{o}C under argon atmosphere was slowly added diisobutyl aluminum hydride (1 M) in toluene (1.2 equivalents) and the resulting mixture was stirred at -70 ^{o}C for 1-2 hours. Methanol (10 mL) was added slowly at -70 ^{o}C and the solution was allowed to warm to room temperature. The reaction mixture was washed with 1N aqueous HCl and extracted with CH_2Cl_2 (2 x 20 mL). The combined organic fractions were dried over anhydrous Na_2SO_4 and evaporated in *vacuuo*. The crude aldehyde was used for the next step without further purification.

Method 3

Aldehyde (1 equivalent) and amine (1.5 equivalents) were mixed in dry MeOH at room temperature under argon atmosphere. The reaction mixture was stirred at room temperature and progress of the reaction was monitored by TLC. After the complete formation of aldimine (3-5 h), $NaBH_4$ (1.5 equivalents) was added slowly at room temperature. The reaction mixture was stirred for 15 more minutes and quenched with 1N NaOH. The product was extracted with CH_2Cl_2 (3 x 20 mL) and dried over anhydrous Na_2SO_4. The solvent was evaporated in *vacuuo* and purified by column chromatography to afford sulindac amine as yellow viscous liquid.

Method 4

Aldehyde (1 equivalent) and amine (1.5 equivalents) were mixed in dry 1,2-dichloroethane under argon atmosphere and then treated with sodium triacetoxyborohydride (1.5 equivalents). The reaction mixture was stirred at room temperature until the complete disappearance of aldehyde (3-5 h). The reaction mixture was quenched with aqueous saturated $NaHCO_3$ and the product was extracted with CH_2Cl_2 (3 x 20 mL). The combined organic fractions were dried over anhydrous Na_2SO_4 and evaporated in *vacuuo*. The product was purified by column chromatography to afford sulindac amine as yellow viscous liquid.

Method 5

To a solution of sulindac sulfide (1 equivalent) in THF at 0 ^{o}C under nitrogen atmosphere, was added a solution of borane in THF (1.2 equivalents) and the reaction mixture stirred in the cold for 30 minutes, then at room temperature for 2 h. Water was slowly added to the reaction mixture and extracted with CH_2Cl_2 (3 x 20 mL). The crude alcohol was purified by flash column chromatography. To a solution of the above alcohol and tetrabutylammonium iodide (2 equivalents) in pyridine (2.2 equivalents) and CH_2Cl_2 was slowly added trifluoromethane sulfonic anhydride (1.8 equivalents) at -78 ^{o}C and stirred for 15 minutes, then at room temperature for 1 h. It was then diluted with CH_2Cl_2 (50 mL) and washed successively with 10% aqueous sodium thiosulfate, 1N aqueous HCl, saturated $NaHCO_3$, and brine. The crude residue from the evaporation of the organic phase was chromatographed to obtain sulindac iodide. The above iodide compound was refluxed with sodium azide (1.5 equivalents) in CH_3CN for 10 h. PPh_3 (1 equivalent) was added to the above solution at room temperature and the reaction mixture was stirred under nitrogen atmosphere for 3 hours. CH_2Cl_2 (50 mL) was added to the reaction mixture and washed with saturated $NaHCO_3$. Solvent was removed under reduced pressure and the product was purified by flash column chromatography to provide sulindac amine as yellow viscous liquid.

Method 6

O-(Benzotriazol-1-yl)-N,N,N',N'-tetramethyluronium hexafluoro phosphate, (HBTU) (1.2 equivalents) was added to a solution of sulindac sulfide amine (1 equivalent), 2-furoic acid (1.5 equivalents) and Et_3N (2 equivalents) in dry acetonitrile (10 mL) at room temperature under argon atmosphere. The reaction mixture was stirred at room temperature for 2 h. Solvent was evaporated under reduced pressure and the crude product was purified by flash column chromatography (60-200 mesh) to afford amide in excellent yield.

Method 7

Oxalyl chloride was added to a solution of sulindac (1.0 equivalent) in CH_2Cl_2 (50 mL) followed by 2 drops of DMF. The resulted reaction mixture was stirred at room temperature for 1 h. Solvent was removed *in vacuuo* and the crude acid chloride was used in the next step without any further purification. Crude acid chloride was suspended in CCl_4 (25 mL) and was added trimethylsilyl azide (1.5 equivalents) at room temperature. The reaction mixture was

stirred at room temperature for 15 min, and then slowly heated while stirring until the evolution of nitrogen ceased. Solvent was removed under reduced pressure to give the isocyanate as a viscous yellow liquid. To the crude isocyanate in acetic acid (80 mL) was slowly added conc. HCl (20 mL). The reaction mixture was heated on a steam bath at 50 °C for 30 min. The reaction mixture was diluted with cold H_2O (100 mL) and filtered. The isolated solid was washed with water and then ether to give sulindac methaneamine as the hydrochloride salt.

Method 8

(2-(7-Aza-1H-benzotriazole-1-yl)-1,1,3,3-tetramethyluronium hexafluorophosphate), (HATU) (1.2 equivalents) was added to a solution of sulindac amine (1 equivalent), the appropriate acid (1.5 equivalents) and DIEA (2 equivalents) in dry acetonitrile (10 mL) at room temperature under argon atmosphere. The reaction mixture was stirred at room temperature for 1-2 h. Solvent was evaporated off under reduced pressure and the crude product was purified by Teledyne Isco Combiflash Rf purification machine to provide amide in excellent yield.

Method 9

To a cold solution of amine (1 equivalent) and N-methyl imidazole (2 equivalents) in dry pyridine (4 mL) was added the appropriate sulfonyl chloride (1.3 equivalents). The reaction mixture was stirred at 0 °C for 3-6 hours. The solvent was removed under reduced pressure, and the crude product was purified by flash column chromatography or using a Teledyne Isco Combiflash Rf purification machine to provide the desired sulfonamide in excellent yield.

3. ANALYTICAL DATA

3.1. (Z)-N-Benzyl-2-(5-Fluoro-2-Methyl-1-(4-(Methylthio) Benzylidene)-1H-Inden-3-yl)Ethanamine (7)

By following methods 1-3, the title compound **7** was obtained as a yellow viscous liquid in 74% (LCMS purity: 100%) yield. ESI-MS m/z: 416.20 $[M+H]^+$.^{1}H NMR ($CDCl_3$, 300 MHz): δ 7.43 (2H, d, J = 8.1 Hz, 3'-H, 5'-H), 7.35-7.21 (8H, m, 2'-H, 6'-H, 7-H, Ph-H), 7.06 (1H, s, 10-H), 6.82 (1H, dd, J = 2.4 Hz, 9.0 Hz, 4-H), 6.56 (1H, td, J = 2.4 Hz, 9.3 Hz, 6-H), 3.83 (2H, s, -CH_2-Ph), 2.89-2.75 (4H, m, -CH_2-CH_2-NH), 2.54 (3H, s, -SCH_3), 2.16 (3H, s, 2-CH_3). HRMS calcd for $[C_{27}H_{26}FNS+H]^+$: 416.18428, Found: 416.18452.

3.2. (Z)-2-(5-Fluoro-2-Methyl-1-(4-(Methylthio) Benzylidene)-1H-inden-3-yl)-N-(Furan-2-ylmethyl)Ethanamine (8)

By following methods 1-3, the title compound **8** was obtained as a yellow viscous liquid in 68% (LCMS purity: 95.8%) yield. ESI-MS m/z: 406.23 $[M+H]^+$.^{1}H NMR ($CDCl_3$, 300 MHz): δ 7.44 (2H, d, J = 8.4 Hz, 3'-H, 5'-H), 7.36-7.26 (4H, m, 2'-H, 6'-H, 7-H, 5''-H), 7.06 (1H, s, 10-H), 6.84 (1H, dd, J = 2.4 Hz, 9.0 Hz, 4-H), 6.59 (1H, td, J = 2.4 Hz, 9.3 Hz, 6-H), 6.31 (1H, dd, J = 1.8 Hz, 3.3 Hz, 4''-H), 6.16 (1H, dd, J = 0.6 Hz, 3.0 Hz, 3''-H), 3.82 (2H, s, -CH_2-Furan), 2.86-2.73 (4H, m, -CH_2-CH_2-NH), 2.54 (3H, s, -SCH_3), 2.16 (3H, s, 2-CH_3). HRMS calcd for $[C_{25}H_{24}FNOS+H]^+$: 406.16354, Found: 406.16388.

3.3. (Z)-2-(5-Fluoro-2-Methyl-1-(4-(Methylthio) Benzylidene)-1H-Inden-3-yl)-N-(2-(Piperidin-1-yl) Ethyl) Ethanamine (9)

By following methods 1-3, the title compound **9** was obtained as a yellow viscous liquid in 55% (LCMS purity: 94.6%) yield. ESI-MS m/z: 437.32 $[M+H]^+$.^{1}H NMR ($CDCl_3$, 300 MHz): δ 7.43 (2H, d, J = 8.4 Hz, 3'-H, 5'-H), 7.36 (1H, dd, J = 5.4 Hz, 8.4 Hz, 7-H), 7.29 (2H, d, J = 8.4 Hz, 2'-H, 6'-H), 7.09 (1H, s, 10-H), 6.87 (1H, dd, J = 2.4 Hz, 9.0 Hz, 4-H), 6.60 (1H, td, J = 2.4 Hz, 9.0 Hz, 6-H), 2.88-2.77 (6H, m, -CH_2-CH_2-NH-CH_2-), 2.54 (3H, s, -SCH_3), 2.51 (2H, t, J = 6.0 Hz, NH-CH_2-C$\underline{H_2}$), 2.38 (4H, t, J = 5.1 Hz, 2''-H, 6''-H), 2.19 (3H, s, 2-CH_3), 1.51-1.48 (4H, m, 3''-H, 5''-H), 1.41-1.35 (2H, m, 4''-H). HRMS calcd for $[C_{27}H_{33}FN_2S+H]^+$: 437.24212, Found: 437.24195.

3.4. (Z)-N1-(2-(5-Fluoro-2-Methyl-1-(4-(Methylthio) Benzylidene)-1H-Inden-3-yl)Ethyl)-N2,N2-Dimethylethane-1,2-Diamine (10)

By following methods 1-3, the title compound **10** was obtained as a yellow viscous liquid in 54% (LCMS purity: 100%) yield. ESI-MS m/z: 397 $[M+H]^+$.^{1}H NMR ($CDCl_3$, 300 MHz): δ 7.43 (2H, d, J = 8.1 Hz, 2'-H, 6'-H), 7.34 (1H, dd, J = 5.1 Hz, 8.4 Hz, 7-H), 7.28 (2H, d, J = 8.4 Hz, 3'-H, 5'-H), 7.07 (1H, s, 10-H), 6.86 (1H, dd, J = 2.4 Hz, 9.3 Hz, 4-H), 6.56 (1H, td, J = 2.4 Hz, 9.3 Hz, 6-H), 2.86-2.72 (6H, m, -CH_2-CH_2-NH-CH_2-), 2.54 (3H, s, -SCH_3), 2.43 (2H, t, J

= 6.3 Hz, C$\underline{H}_2$-N(CH_3)$_2$), 2.22 (6H, s, -N(CH_3)$_2$), 2.17 (3H, s, 2-CH_3). HRMS calcd for [$C_{24}H_{29}FN_2S$+H]$^+$: 397.21082, Found: 397.21066.

3.5. (Z)-N-benzyl-2-(5-Fluoro-2-Methyl-1-(4-(Methylsulfinyl) Benzylidene)-1H-Inden-3-yl) Ethanamine (11)

By following methods 1, 2, and 4, the title compound **11** was obtained as a yellow viscous liquid in 61% (LCMS purity: 97.6%) yield. ^{1}H NMR ($CDCl_3$, 300 MHz): δ 7.72-7.63 (4H, m, 2'-H, 3'-H, 5'-H, 6'-H), 7.32-7.22 (5H, m, Ph-H), 7.15 (1H, dd, *J* = 5.4 Hz, 8.4 Hz, 7-H), 7.08 (1H, s, 8-H), 6.85 (1H, dd, *J* = 2.7 Hz, 9.3 Hz, 4-H), 6.57 (1H, ddd, *J* = 2.4 Hz, 9.3 Hz, 11.1 Hz, 6-H), 3.84 (2H, s, -CH_2-Ph), 2.90-2.75 (4H, m, -CH_2-CH_2-NH), 2.80 (3H, s, -$SOCH_3$), 2.17 (3H, s, 2-CH_3). HRMS calcd for [$C_{27}H_{26}FNOS$+H]$^+$: 432.17919, Found: 432.17990.

3.6. (Z)-N-Benzyl-2-(5-Fluoro-2-Methyl-1-(4-(Methylsulfonyl) Benzylidene)-1H-Inden-3-yl)Ethanamine (12)

By following methods 1, 2, and 4, the title compound **12** was obtained as a yellow solid in 51% (HPLC purity: 98.3%) yield. mp 96-98 °C. ^{1}H NMR ($CDCl_3$, 400 MHz): δ 7.99 (2H, d, J = 8.4 Hz, 3'-H, 5'-H), 7.68 (2H, d, J = 8.4 Hz, 2'-H, 6'-H), 7.34-7.22 (5H, m, Ph-H), 7.08 (1H, dd, J = 5.2 Hz, 8.4 Hz, 7-H), 7.04 (1H, s, 8-H), 6.83 (1H, dd, J = 2.4 Hz, 8.8 Hz, 4-H), 6.55 (1H, td, J = 2.4 Hz, 8.8 Hz, 6-H), 3.84 (2H, s, -CH_2-Ph), 3.13 (3H, s, -SO_2CH_3), 2.87 (2H, t, J = 6.8 Hz, -CH_2-C$\underline{H}_2$-NH), 2.77 (2H, t, J = 6.4 Hz, -C$\underline{H}_2$-CH_2-NH), 2.16 (3H, s, 2-CH_3). HRMS calcd for [$C_{27}H_{26}FNO_2S$+H]$^+$: 448.17410, Found: 448.17467.

3.7. (Z)-2-(5-Fluoro-2-Methyl-1-(4-(methylthio)benzylidene)-1H-inden-3-yl)ethanamine (13)

By following method 5, the title compound **13** was obtained as a yellow viscous liquid in 57% (LCMS purity: 95.8%) yield. ESI-MS m/z: 326.18 [M+H]$^+$.^{1}H NMR ($CDCl_3$, 300 MHz): δ 7.43 (2H, d, J = 8.1 Hz, 3'-H, 5'-H), 7.34 (1H, dd, J = 5.4 Hz, 8.4 Hz, 7-H), 7.28 (2H, d, J = 8.1 Hz, 2'-H, 6'-H), 7.08 (1H, s, 10-H), 6.84 (1H, dd, J = 2.4 Hz, 9.0 Hz, 4-H), 6.56 (1H, td, J = 2.4 Hz, 9.0 Hz, 6-H), 2.94 (2H, t, J = 6.3 Hz, C$\underline{H}_2$-NH_2), 2.71 (2H, t, J = 6.9 Hz, 3-CH_2), 2.54 (3H, s, -SCH_3), 2.18 (3H, s, 2-CH_3). HRMS calcd for [$C_{20}H_{20}FNS$+H]$^+$: 326.13732, Found: 326.13805.

3.8. (Z)-N-(2-(5-Fluoro-2-Methyl-1-(4-(Methylthio) Benzylidene)-1H-Inden-3-yl) Ethyl) Furan-2-Carboxamide (14)

By following methods 5, and 6, the title compound **14** was obtained as a yellow solid in 86% (LCMS purity: 97.5%) yield. mp 168-170 °C. ESI-MS m/z: 420.24 [M+H]$^+$.^{1}H NMR ($CDCl_3$, 300 MHz): δ 7.45 (2H, d, J = 8.1 Hz, 3'-H, 5'-H), 7.40 (1H, dd, J = 0.6 Hz, 1.5 Hz, 3''-H), 7.37 (1H, dd, J = 5.1 Hz, 8.4 Hz, 7-H), 7.29 (2H, d, J = 8.4 Hz, 2'-H, 6'-H), 7.12 (1H, dd, J = 0.9 Hz, 3.6 Hz, 5''-H), 7.10 (1H, s, 10-H), 6.91 (1H, dd, J = 2.4 Hz, 9.0 Hz, 4-H), 6.58 (1H, td, J = 2.1 Hz, 9.3 Hz, 6-H), 6.49 (1H, dd, J = 1.5 Hz, 3.3 Hz, 4''-H), 6.46 (1H, bs, NH), 3.62 (2H, q, J = 6.6 Hz, C$\underline{H}_2$-NHCO), 2.87 (2H, t, J = 6.9 Hz, 3-CH_2), 2.55 (3H, s, SCH_3), 2.16 (3H, s, 2-CH_3). HRMS calcd for [$C_{25}H_{22}FNO_2S$+Na]$^+$: 442.12475, Found: 442.12546.

3.9. (Z)-(5-Fluoro-2-Methyl-1-(4-(Methylthio) Benzylidene)-1H-Inden-3-yl) Methanamine (16)

By following method 7, the title compound **16** was obtained as a yellow viscous liquid in 70% (HPLC purity: 98.7%) yield. ^{1}H NMR (DMSO, 400 MHz): δ 7.48 (2H, d, J = 8.4 Hz, 2'-H, 6'-H), 7.35 (2H, d, J = 8.4 Hz, 3'-H, 5'-H), 7.32 (1H, dd, J = 5.6 Hz, 8.4 Hz, 7-H), 7.24 (1H, s, 10-H), 7.20 (1H, dd, J = 2.0 Hz, 9.2 Hz, 4-H), 6.71 (1H, td, J = 2.0 Hz, 9.2 Hz, 6-H), 3.65 (2H, s, C$\underline{H}_2$-NH_2), 2.53 (3H, s, -SCH_3), 2.15 (3H, s, 2-CH_3), 1.64 (2H, s, NH_2). HRMS calcd for [$C_{19}H_{18}FNS$-NH_4+H]$^+$: 295.09513, Found: 295.09514.

3.10. (Z)-(5-Fluoro-2-Methyl-1-(3,4,5-Trimethoxybenzylidene)-1H-Inden-3-yl) Methanamine (17)

By following method 7, the title compound **17** was obtained as a yellow solid in 88% (HPLC purity: 90.5%) yield. mp 108-110 °C. ^{1}H NMR ($CDCl_3$, 400 MHz): δ 7.45 (1H, dd, J = 4.8 Hz, 8.0 Hz, 7-H), 7.14 (1H, s, 10-H), 6.96 (1H, dd, J = 2.4 Hz, 8.4 Hz, 4-H), 6.74 (2H, s, 2'-H, 6'-H), 6.61 (1H, td, J = 2.8 Hz, 9.6 Hz, 6-H), 3.93 (3H, s, 4'-OCH_3), 3.84 (6H, s, 3'-OCH_3, 5'-OCH_3), 3.82 (2H, s, C$\underline{H}_2$-NH_2), 2.73 (2H, s, NH_2), 2.21 (3H, s, 2-CH_3). HRMS calcd for [$C_{21}H_{22}FNO_3$-NH_4+H]$^+$: 339.13910, Found: 339.13935.

3.11. (Z)-N-((5-Fluoro-2-Methyl-1-(4-(Methylthio) Benzylidene)-1H-Inden-3-yl) Methyl)-2-Phenylacetamide (18)

By following methods 7-8, the title compound **18** was obtained as a yellow solid in 74% (HPLC purity: 100%)

yield. mp 164-166 °C. ^{1}H NMR ($CDCl_3$, 400 MHz): δ 7.42 (2H, d, J = 8.4 Hz, 2'-H, 6'-H), 7.35 (1H, dd, J = 4.8 Hz, 8.4 Hz, 7-H), 7.32-7.23 (7H, m, 3'-H, 5'-H, Ph-H), 7.13 (1H, s, 10-H), 6.76 (1H, dd, J = 2.4 Hz, 8.8 Hz, 4-H), 6.58 (1H, td, J = 2.4 Hz, 9.2 Hz, 6-H), 5.39 (1H, s, 3-CH_2-N$\underline{H}$), 4.38 (2H, d, J = 5.2 Hz, 3-CH_2), 3.60 (2H, s, CH_2Ph), 2.54 (3H, s, -SCH_3), 2.15 (3H, s, 2-CH_3). HRMS calcd for $[C_{27}H_{24}FNOS+H]^+$: 430.16354, Found: 430.16367.

3.12. (Z)-N-((5-Fluoro-2-Methyl-1-(4-(Methylthio) Benzylidene)-1H-Inden-3-yl) Methyl)furan-2-Carboxamide (19)

By following methods 7-8, the title compound **19** was obtained as a yellow solid in 53% (HPLC purity: 99.2%) yield. mp 173-176 °C. ^{1}H NMR ($CDCl_3$, 400 MHz): δ 7.44 (2H, d, J = 8.4 Hz, 2'-H, 6'-H), 7.40-7.37 (2H, m, 5''-H, 7-H), 7.29 (2H, d, J = 8.8 Hz, 3'-H, 5'-H), 7.19 (1H, s, 10-H), 7.15 (1H, dd, J = 0.8 Hz, 3.6 Hz, 3''-H), 6.96 (1H, dd, J = 2.4 Hz, 8.8 Hz, 4-H), 6.60 (1H, td, J = 2.4 Hz, 9.2 Hz, 6-H), 6.49 (1H, dd, J = 1.6 Hz, 3.6 Hz, 4''-H), 6.33 (1H, bs, NH), 4.58 (2H, d, J = 5.2 Hz, 3-CH_2), 2.54 (3H, s, SCH_3), 2.27 (3H, s, 2-CH_3). HRMS calcd for $[C_{24}H_{20}FNO_2S+H]^+$: 406.12715, Found: 406.12701.

3.13. (S,Z)-2-Amino-N-((5-Fluoro-2-Methyl-1-(4-(Methylthio) Benzylidene)-1H-Inden-3-yl) Methyl)-3-(1H-Imidazol-4-yl) Propanamide (20)

By following methods 7-8 using N-Boc-L-histidine and acidic removal of the Boc protecting group, the title compound **20** was obtained as a yellow solid in 33% (HPLC purity: 100%) overall yield. mp 99-101 °C. ^{1}H NMR (DMSO, 400 MHz): δ 11.75 (1H, bs, 1''-H), 8.21 (1H, bt, NHCO), 7.50 (2H, d, J = 8.0 Hz, 2'-H, 6'-H), 7.49 (1H, s, 2''-H), 7.36-7.32 (3H, m, 7-H, 3'-H, 5'-H), 7.31 (1H, s, 10-H), 7.14 (1H, dd, J = 2.4 Hz, 9.2 Hz, 4-H), 6.77 (1H, s, 5''-H), 6.73 (1H, td, J = 2.4 Hz, 9.2 Hz, 6-H), 4.25 (2H, d, J = 5.6 Hz, 3-CH_2), 3.43-3.25 (2H, m, $C\underline{H}_2$-Im), 2.86 (1H, dd, J = 3.6 Hz, 14.0 Hz, $C\underline{H}$-NH_2), 2.53 (3H, s, -SCH_3), 2.21 (3H, s, 2-CH_3). HRMS calcd for $[C_{25}H_{25}FN_4OS+H]^+$: 449.18059, Found: 449.18071.

3.14. (Z)-2-Amino-N-((5-Fluoro-2-Methyl-1-(4-(Methylthio) Benzylidene)-1H-Inden-3-yl) Methyl) Acetamide (21)

By following methods 7-8 using Boc-glycine and acidic removal of the Boc protecting group, the title compound **21** was obtained as a yellow solid in 84% (HPLC purity: 96.4%) overall yield. mp 122-124 °C. ^{1}H NMR ($CDCl_3$, 400 MHz): δ 7.44 (2H, d, J = 8.8 Hz, 2'-H, 6'-H), 7.38 (1H, dd, J = 5.2 Hz, 8.4 Hz, 7-H), 7.30 (1H, t, J = 4.8 Hz, 3-CH_2-N$\underline{H}$), 7.29 (2H, d, J = 8.4 Hz, 3'-H, 5'-H), 7.16 (1H, s, 10-H), 6.92 (1H, dd, J = 2.4 Hz, 9.2 Hz, 4-H), 6.59 (1H, td, J = 2.8 Hz, 8.8 Hz, 6-H), 4.44 (2H, d, J = 5.2 Hz, 3-CH_2), 3.39 (2H, s, $C\underline{H}_2$-NH_2), 2.55 (3H, s, -SCH_3), 2.24 (3H, s, 2-CH_3), 1.35 (2H, s, NH_2). HRMS calcd for $[C_{21}H_{21}FN_2OS+H]^+$: 369.14314, Found: 369.14299.

3.15. (S,Z)-2-Amino-N-((5-Fluoro-2-Methyl-1-(4-(Methylthio) Benzylidene)-1H-Inden-3-yl) Methyl)-3-Hydroxypropanamide (22)

By following methods 7-8 using Boc-L-serine and acidic removal of the Boc protecting group, the title compound **22** was obtained as a yellow solid in 83% (HPLC purity: 99.2%) overall yield. mp 146-148 °C. ^{1}H NMR ($CDCl_3$, 400 MHz): δ 7.47 (1H, s, 3-CH_2-N$\underline{H}$), 7.44 (2H, d, J = 8.4 Hz, 2'-H, 6'-H), 7.38 (1H, dd, J = 5.2 Hz, 8.4 Hz, 7-H), 7.29 (2H, d, J = 8.4 Hz, 3'-H, 5'-H), 7.17 (1H, s, 10-H), 6.90 (1H, dd, J = 2.4 Hz, 9.2 Hz, 4-H), 6.60 (1H, td, J = 2.4 Hz, 8.8 Hz, 6-H), 4.47-4.36 (2H, m, 3-CH_2), 3.91 (1H, dd, J = 5.2 Hz, 11.2 Hz, CH_{2a}-OH), 3.73 (1H, dd, J = 6.0 Hz, 10.8 Hz, CH_{2b}-OH), 3.47 (1H, t, J = 5.6 Hz, CH), 2.72 (1H, bs, -OH), 2.55 (3H, s, -SCH_3), 2.23 (3H, s, 2-CH_3). HRMS calcd for $[C_{22}H_{23}FN_2O_2S+H]^+$: 399.15370, Found: 399.15427.

3.16. (S,Z)-2-Amino-N-((5-Fluoro-2-Methyl-1-(4-(Methylthio) Benzylidene)-1H-Inden-3-yl) Methyl)-4-Methylpentanamide (23)

By following methods 7-8 using Boc-L-leucine and acidic removal of the Boc protecting group, the title compound **23** was obtained as a yellow viscous liquid in 86% (HPLC purity: 94.3%) overall yield. ^{1}H NMR ($CDCl_3$, 400 MHz): δ 7.44 (2H, d, J = 8.0 Hz, 2'-H, 6'-H), 7.37 (1H, dd, J = 5.2 Hz, 8.0 Hz, 7-H), 7.34 (1H, s, 3-CH_2-N$\underline{H}$), 7.29 (2H, d, J = 8.4 Hz, 3'-H, 5'-H), 7.16 (1H, s, 10-H), 6.90 (1H, dd, J = 2.4 Hz, 8.8 Hz, 4-H), 6.59 (1H, td, J = 2.4 Hz, 9.2 Hz, 6-H), 4.45-4.35 (2H, m, 3-CH_2), 3.43 (1H, dd, J = 3.6 Hz, 10.0 Hz, COCH), 2.55 (3H, s, -SCH_3), 2.23 (3H, s, 2-CH_3), 1.79-1.29 (3H, m, $C\underline{H}_2C\underline{H}(CH_3)_2$), 0.96 (3H, d, J = 6.0 Hz, $CH(C\underline{H}_3)_2$), 0.93 (3H, d, J = 6.4 Hz, $CH(C\underline{H}_3)_2$). HRMS calcd for $[C_{25}H_{29}FN_2OS+H]^+$: 425.20574, Found: 425.20625.

3.17. (S,Z)-2-Amino-N-((5-Fluoro-2-Methyl-1-(4-(Methylthio) Benzylidene)-1H-inden-3-yl)methyl)-3-Methylbutanamide (24)

By following methods 7-8 using Boc-L-valine and acidic removal of the Boc protecting group, the title compound **24** was obtained as a yellow solid in 83% (HPLC purity: 100%) overall yield. mp 133-135 °C. ^{1}H NMR ($CDCl_3$, 400 MHz): δ 7.44 (2H, d, J = 8.0 Hz, 2'-H, 6'-H), 7.37 (1H, dd, J = 5.2 Hz, 8.4 Hz, 7-H), 7.35 (1H, s, 3-CH_2-N$\underline{H}$), 7.29 (2H, d, J = 8.8 Hz, 3'-H, 5'-H), 7.16 (1H, s, 10-H), 6.92 (1H, dd, J = 2.8 Hz, 8.8 Hz, 4-H), 6.59 (1H, td, J = 2.4 Hz, 8.8 Hz, 6-H), 4.41 (2H, d, J = 5.6 Hz, 3-CH_2), 3.27 (1H, d, J = 3.6 Hz, COCH), 2.55 (3H, s, -SCH_3), 2.42-2.34 (1H, m, C$\underline{H}$(CH_3)$_2$), 2.23 (3H, s, 2-CH_3), 0.99 (3H, d, J = 7.2 Hz, CH(C$\underline{H}_3$)$_2$), 0.90 (3H, d, J = 7.2 Hz, CH(C$\underline{H}_3$)$_2$). HRMS calcd for $[C_{24}H_{27}FN_2OS+H]^+$: 411.19009, Found: 411.19001.

3.18. (S,Z)-N-((5-Fluoro-2-Methyl-1-(4-(Methylthio)benzylidene)-1H-Inden-3-yl) Methyl) Pyrrolidine-2-Carboxamide (25)

By following methods 7-8 using Boc-L-proline and acidic removal of the Boc protecting group, the title compound **25** was obtained as a yellow viscous liquid in 80% (HPLC purity: 93.7%) overall yield.^{1}H NMR ($CDCl_3$, 400 MHz): δ 7.66 (1H, s, 3-CH_2-N$\underline{H}$), 7.44 (2H, d, J = 8.4 Hz, 2'-H, 6'-H), 7.36 (1H, dd, J = 5.2 Hz, 8.0 Hz, 7-H), 7.29 (2H, d, J = 8.4 Hz, 3'-H, 5'-H), 7.15 (1H, s, 10-H), 6.89 (1H, dd, J = 2.8 Hz, 9.2 Hz, 4-H), 6.58 (1H, td, J = 2.4 Hz, 8.8 Hz, 6-H), 4.43-4.34 (2H, m, 3-CH_2), 3.78 (1H, dd, J = 5.6 Hz, 9.6 Hz, 2''-H), 2.96 (2H, dt, J = 7.2 Hz, 10.0 Hz, 5''-H_a), 2.81 (2H, dt, J = 6.4 Hz, 10.0 Hz, 5''-H_b), 2.54 (3H, s, -SCH_3), 2.22 (3H, s, 2-CH_3), 2.19-1.63 (4H, m, 3''-H, 4''-H). HRMS calcd for $[C_{24}H_{25}FN_2OS+H]^+$: 409.17444, Found: 409.17518.

3.19. (Z)-N-((5-Fluoro-2-Methyl-1-(3,4,5-Trimethoxybenzylidene)-1H-Inden-3-yl) Methyl)-2-Phenylacetamide (26)

By following methods 7-8, the title compound **26** was obtained as a yellow solid in 73% (HPLC purity: 100%) yield. mp 184-187 °C. ^{1}H NMR ($CDCl_3$, 400 MHz): δ 7.42 (1H, dd, J = 5.2 Hz, 8.4 Hz, 7-H), 7.34-7.23 (5H, m, Ph-H), 7.13 (1H, s, 10-H), 6.77 (1H, dd, J = 2.4 Hz, 8.8 Hz, 4-H), 6.72 (2H, s, 2'-H, 6'-H), 6.60 (1H, td, J = 2.4 Hz, 8.4 Hz, 6-H), 5.41 (1H, bs, NH), 4.38 (2H, d, J = 5.6 Hz, 3-CH_2), 3.92 (3H, s, 4'-OCH_3), 3.83 (6H, s, 3'-OCH_3, 5'-OCH_3), 3.60 (2H, s, CH_2Ph), 2.16 (3H, s, 2-CH_3). HRMS calcd for $[C_{29}H_{28}FNO_4+H]^+$: 474.20751, Found: 474.20794.

3.20. (Z)-N-((5-Fluoro-2-Methyl-1-(3,4,5-Trimethoxybenzylidene)-1H-Inden-3-yl) Methyl)furan-2-Carboxamide (27)

By following methods 7-8, the title compound **27** was obtained as a yellow solid in 69% (HPLC purity: 100%) yield. mp 192-194 °C. ^{1}H NMR ($CDCl_3$, 400 MHz): δ 7.46 (1H, dd, J = 5.2 Hz, 8.4 Hz, 7-H), 7.39 (1H, dd, J = 0.8 Hz, 2.0 Hz, 5''-H), 7.19 (1H, s, 10-H), 7.16 (1H, dd, J = 0.8 Hz, 3.6 Hz, 3''-H), 6.98 (1H, dd, J = 2.4 Hz, 9.2 Hz, 4-H), 6.75 (2H, s, 2'-H, 6'-H), 6.62 (1H, td, J = 2.0 Hz, 8.8 Hz, 6-H), 6.50 (1H, dd, J = 2.0 Hz, 3.6 Hz, 4''-H), 6.34 (1H, bs, NH), 4.58 (2H, d, J = 5.6 Hz, 3-CH_2), 3.93 (3H, s, 4'-OCH_3), 3.84 (6H, s, 3'-OCH_3, 5'-OCH_3), 2.28 (3H, s, 2-CH_3). HRMS calcd for $[C_{26}H_{24}FNO_5+H]^+$: 450.17113, Found: 450.17185.

3.21. (Z)-N-((5-Fluoro-2-Methyl-1-(3,4,5-Trimethoxybenzylidene)-1H-Inden-3-yl) Methyl)acetamide (28)

By following methods 7-8, the title compound **28** was obtained as a yellow solid in 82% (HPLC purity: 100%) yield. mp 183-185 °C. ^{1}H NMR ($CDCl_3$, 400 MHz): δ 7.46 (1H, dd, J = 5.2 Hz, 8.0 Hz, 7-H), 7.17 (1H, s, 10-H), 6.92 (1H, dd, J = 2.4 Hz, 8.4 Hz, 4-H), 6.74 (2H, s, 2'-H, 6'-H), 6.63 (1H, td, J = 2.0 Hz, 8.4 Hz, 6-H), 5.41 (1H, bs, NH), 4.41 (2H, d, J = 5.6 Hz, 3-CH_2), 3.93 (3H, s, 4'-OCH_3), 3.84 (6H, s, 3'-OCH_3, 5'-OCH_3), 2.23 (3H, s, 2-CH_3), 2.00 (3H, s, $COCH_3$). HRMS calcd for $[C_{23}H_{24}FNO_4+H]^+$: 420.15887, Found: 420.15909.

3.22. (Z)-2-Amino-N-((5-Fluoro-2-Methyl-1-(3,4,5-Trimethoxybenzylidene)-1H-Inden-3-yl) Methyl) Acetamide (29)

By following methods 7-8 using Boc-glycine and acidic removal of the Boc protecting group, the title compound **29** was obtained as a yellow solid in 44% (HPLC purity: 90.5%) overall yield. mp 58-60 °C. ^{1}H NMR ($CDCl_3$, 400 MHz): δ 7.45 (1H, dd, J = 4.8 Hz, 8.4 Hz, 7-H), 7.30 (1H, bs, NHCO), 7.17 (1H, s, 10-H), 6.94 (1H, dd, J = 2.4 Hz, 8.8 Hz, 4-H), 6.74 (2H, s, 2'-H, 6'-H), 6.62 (1H, td, J = 2.4 Hz, 9.2 Hz, 6-H), 4.44 (2H, d, J = 5.2 Hz, 3-CH_2), 3.93 (3H, s, 4'-OCH_3), 3.84 (6H, s, 3'-OCH_3, 5'-OCH_3), 3.40 (2H, s, COC$\underline{H}_2$$NH_2$), 2.24 (3H, s, 2-$CH_3$), 1.43 (2H, bs, CH_2N$\underline{H}_2$). HRMS

calcd for $[C_{23}H_{25}FN_2O_4+H]^+$: 413.18711, Found: 413.18742.

3.23. (S,Z)-2-Amino-N-((5-Fluoro-2-Methyl-1-(3,4,5-Trimethoxybenzylidene)-1H-Inden-3-yl)methyl)-3-Hydroxypropanamide (30)

By following methods 7-8 using Boc-L-serine and acidic removal the Boc protecting group, the title compound **30** was obtained as a yellow solid in 48% (HPLC purity: 100%) overall yield. mp 118-120 °C. ^{1}H NMR ($CDCl_3$, 400 MHz): δ 7.48 (1H, bs, NHCO), 7.45 (1H, dd, J = 5.2 Hz, 8.4 Hz, 7-H), 7.17 (1H, s, 10-H), 6.91 (1H, dd, J = 2.4 Hz, 8.8 Hz, 4-H), 6.74 (2H, s, 2'-H, 6'-H), 6.62 (1H, td, J = 2.4 Hz, 9.2 Hz, 6-H), 4.48-4.37 (2H, m, 3-CH_2), 3.93 (3H, s, 4'-OCH_3), 3.91 (1H, dd, J = 6.0 Hz, 11.2 Hz, $C\underline{H}_{2a}OH$), 3.84 (6H, s, 3'-OCH_3, 5'-OCH_3), 3.73 (1H, dd, J = 6.0 Hz, 11.2 Hz, $C\underline{H}_{2b}OH$), 3.47 (1H, t, J = 5.2 Hz, CH), 2.65 (1H, bs, NH), 2.24 (3H, s, 2-CH_3), 1.54 (2H, bs, NH_2). HRMS calcd for $[C_{24}H_{27}FN_2O_5+H]^+$: 443.19768, Found: 443.19775.

3.24. (S,Z)-2-Amino-N-((5-Fluoro-1-(3,4,5-Trimethoxybenzylidene)-1H-Inden-3-yl) Methyl)-4-Methylpentanamide (31)

By following methods 7-8 using Boc-L-leucine and acidic removal the Boc protecting group, the title compound **31** was obtained as a yellow solid in 49% (HPLC purity: 92.5%) overall yield. mp 53-55 °C. ^{1}H NMR ($CDCl_3$, 400 MHz): δ 7.44 (1H, dd, J = 5.6 Hz, 8.8 Hz, 7-H), 7.37 (1H, bt, NHCO), 7.16 (1H, s, 10-H), 6.92 (1H, dd, J = 2.8 Hz, 8.8 Hz, 4-H), 6.74 (2H, s, 2'-H, 6'-H), 6.61 (1H, td, J = 2.4 Hz, 9.2 Hz, 6-H), 4.45-4.36 (2H, m, 3-CH_2), 3.93 (3H, s, 4'-OCH_3), 3.84 (6H, s, 3'-OCH_3, 5'-OCH_3), 3.43 (1H, dd, J = 3.6 Hz, 10.0 Hz, $NHCOC\underline{H}$), 2.23 (3H, s, 2-CH_3), 1.78-1.32 (3H, m, $C\underline{H}_2C\underline{H}(CH_3)_2$), 1.50 (2H, bs, NH_2), 0.97 (3H, d, J = 6.4 Hz, $CH(C\underline{H}_3)_2$), 0.94 (3H, d, J = 6.4 Hz, $CH(C\underline{H}_3)_2$). HRMS calcd for $[C_{27}H_{33}FN_2O_4+H]^+$: 469.24971, Found: 469.24934.

3.25. (S,Z)-2-Amino-N-((5-Fluoro-2-Methyl-1-(3,4,5-Trimethoxybenzylidene)-1H-Inden-3-yl) Methyl)-3-Methylbutanamide (32)

By following methods 7-8 using Boc-L-valine and acidic removal the Boc protecting group, the title compound **32** was obtained as a yellow viscous liquid in 45% (HPLC purity: 93.1%) overall yield. ^{1}H NMR ($CDCl_3$, 400 MHz): δ 7.44 (1H, dd, J = 5.6 Hz, 8.8 Hz, 7-H), 7.36 (1H, bt, NHCO), 7.16 (1H, s, 10-H), 6.93 (1H, dd, J = 2.4 Hz, 9.2 Hz, 4-H), 6.74 (2H, s, 2'-H, 6'-H), 6.61 (1H, td, J = 2.4 Hz, 9.2 Hz, 6-H), 4.42 (2H, d, J = 5.6 Hz, 3-CH_2), 3.92 (3H, s, 4'-OCH_3), 3.84 (6H, s, 3'-OCH_3, 5'-OCH_3), 3.27 (1H, d, J = 3.6 Hz, $NHCOC\underline{H}$), 2.42-2.34 (1H, m, $C\underline{H}(CH_3)_2$), 2.23 (3H, s, 2-CH_3), 1.22 (2H, bs, NH_2), 0.99 (3H, d, J = 7.2 Hz, $CH(C\underline{H}_3)_2$), 0.82 (3H, d, J = 6.8 Hz, $CH(C\underline{H}_3)_2$). HRMS calcd for $[C_{26}H_{31}FN_2O_4+H]^+$: 455.23406, Found: 455.23423.

3.26. (S,Z)-N-((5-Fluoro-2-Methyl-1-(3,4,5-Trimethoxybenzylidene)-1H-Inden-3-yl) Methyl)pyrrolidine-2-Carboxamide (33)

By following methods 7-8 Boc-L-proline and acidic removal the Boc protecting group, the title compound **33** was obtained as a yellow solid in 47% (HPLC purity: 91.7%) overall yield. mp 64-66 °C. ^{1}H NMR ($CDCl_3$, 400 MHz): δ 7.66 (1H, bt, NHCO), 7.44 (1H, dd, J = 5.2 Hz, 8.8 Hz, 7-H), 7.16 (1H, s, 10-H), 6.91 (1H, dd, J = 2.0 Hz, 8.8 Hz, 4-H), 6.74 (2H, s, 2'-H, 6'-H), 6.60 (1H, td, J = 2.4 Hz, 9.2 Hz, 6-H), 4.44-4.34 (2H, m, 3-CH_2), 3.93 (3H, s, 4'-OCH_3), 3.84 (6H, s, 3'-OCH_3, 5'-OCH_3), 3.78 (1H, dd, J = 5.6 Hz, 9.6 Hz, 2''-H), 2.99-2.79 (2H, m, 5''-H), 2.23 (3H, s, 2-CH_3), 2.19-1.65 (4H, m, 3''-H, 4''-H). HRMS calcd for $[C_{26}H_{29}FN_2O_4+H]^+$: 453.21841, Found: 453.21821.

3.27. (Z)-N-((5-Fluoro-2-Methyl-1-(4-(methylthio) Benzylidene)-1H-Inden-3-yl)methyl) Methanesulfonamide (34)

By following methods 7 and 9, the title compound **34** was obtained as a yellow solid in 85% (HPLC purity: 100%) yield. mp 159-160 °C. ^{1}H NMR ($CDCl_3$, 400 MHz): δ 7.44 (2H, d, J = 8.4 Hz, 2'-H, 6'-H), 7.42 (1H, dd, J = 5.2 Hz, 8.4 Hz, 7-H), 7.29 (2H, d, J = 8.4 Hz, 3'-H, 5'-H), 7.20 (1H, s, 10-H), 6.97 (1H, dd, J = 2.4 Hz, 8.8 Hz, 4-H), 6.63 (1H, td, J = 2.8 Hz, 9.2 Hz, 6-H), 4.30 (3H, s, 3-$C\underline{H}_2N\underline{H}$), 2.93 (3H, s, -$SO_2CH_3$), 2.55 (3H, s, -$SCH_3$), 2.26 (3H, s, 2-$CH_3$). HRMS calcd for $[C_{20}H_{20}FNO_2S_2+Na]^+$: 412.08117, Found: 412.08150.

3.28. (Z)-N-((5-Fluoro-2-Methyl-1-(4-(Methylthio)benzylidene)-1H-Inden-3-yl) Methyl)ethanesulfonamide (35)

By following methods 7 and 9, the title compound **35** was obtained as a yellow solid in 87% (HPLC purity: 100%) yield. mp 131-133 °C. ^{1}H NMR ($CDCl_3$, 400 MHz): δ 7.44 (2H, d, J = 8.4 Hz, 2'-H, 6'-H), 7.42 (1H, dd, J = 5.2 Hz, 8.4

Hz, 7-H), 7.29 (2H, d, J = 8.8 Hz, 3'-H, 5'-H), 7.20 (1H, s, 10-H), 6.97 (1H, dd, J = 2.4 Hz, 8.8 Hz, 4-H), 6.63 (1H, td, J = 2.4 Hz, 9.2 Hz, 6-H), 4.28 (2H, d, J = 5.6 Hz, 3-CH_2), 4.20 (1H, t, J = 5.2 Hz, NH), 3.03 (2H, q, J = 7.2 Hz, $SO_2C\underline{H}_2CH_3$), 2.55 (3H, s, -SCH_3), 2.25 (3H, s, 2-CH_3), 1.33 (2H, t, J = 7.6 Hz, $SO_2CH_2C\underline{H}_3$). HRMS calcd for $[C_{21}H_{22}FNO_2S_2+Na]^+$: 426.09682, Found: 426.09693.

3.29. (Z)-N-((5-Fluoro-2-Methyl-1-(4-(Methylthio)benzylidene)-1H-Inden-3-yl) Methyl)benzenesulfonamide (36)

By following methods 7 and 9, the title compound **36** was obtained as a yellow solid in 93% (HPLC purity: 100%) yield. mp 141-143 °C. ^{1}H NMR ($CDCl_3$, 400 MHz): δ 7.90-7.87 (2H, m, Ph-H), 7.61-7.48 (3H, m, Ph-H), 7.40 (2H, d, J = 8.4 Hz, 2'-H, 6'-H), 7.33 (1H, dd, J = 5.6 Hz, 8.4 Hz, 7-H), 7.28 (2H, d, J = 8.0 Hz, 3'-H, 5'-H), 7.10 (1H, s, 10-H), 6.59-6.53 (2H, m, 4-H, 6-H), 4.39 (1H, t, J = 4.8 Hz, NH), 4.10 (2H, d, J = 5.6 Hz, 3-CH_2), 2.54 (3H, s, -SCH_3), 2.08 (3H, s, 2-CH_3). HRMS calcd for $[C_{25}H_{22}FNO_2S_2+Na]^+$: 474.09682, Found: 474.09714.

3.30. (Z)-N-((5-Fluoro-2-Methyl-1-(4-(Methylthio)benzylidene)-1H-Inden-3-yl) Methyl)-4-Methylbenzenesul Fonamide (37)

By following methods 7 and 9, the title compound **37** was obtained as a yellow solid in 86% (HPLC purity: 100%) yield. mp 173-175 °C. ^{1}H NMR ($CDCl_3$, 400 MHz): δ 7.76 (2H, d, J = 8.4 Hz, 2''-H, 6''-H), 7.40 (2H, d, J = 8.0 Hz, 2'-H, 6'-H), 7.32 (1H, dd, J = 5.2 Hz, 8.4 Hz, 7-H), 7.31-7.27 (4H, m, 3'-H, 5'-H, 3''-H, 5''-H), 7.11 (1H, s, 10-H), 6.55 (1H, td, J = 2.4 Hz, 9.2 Hz, 6-H), 6.50 (1H, dd, J = 2.4 Hz, 9.2 Hz, 4-H), 4.31 (1H, t, J = 5.2 Hz, NH), 4.08 (2H, d, J = 5.2 Hz, 3-CH_2), 2.54 (3H, s, -SCH_3), 2.43 (3H, s, -4''-CH_3), 2.09 (3H, s, 2-CH_3). HRMS calcd for $[C_{26}H_{24}FNO_2S_2+H]^+$: 488.11247, Found: 488.11239.

3.31. (Z)-N-((5-Fluoro-2-Methyl-1-(4-(Methylthio) Benzylidene)-1H-Inden-3-yl) Methyl)naphthalene-1-Sulfonamide (38)

By following methods 7 and 9, the title compound **38** was obtained as a yellow solid in 85% (HPLC purity: 100%) yield. mp 94-95 °C. ^{1}H NMR ($CDCl_3$, 400 MHz): δ 8.57 (1H, dd, J = 1.2 Hz, 8.8 Hz, 8''-H), 8.27 (1H, dd, J = 1.2 Hz, 7.2 Hz, 2''-H), 8.01 (1H, d, J = 8.4 Hz, 4''-H), 7.89 (1H, dd, J = 1.6 Hz, 8.0 Hz, 5''-H), 7.63-7.46 (3H, m, 3''-H, 6''-H, 7''-H), 7.37 (2H, d, J = 8.0 Hz, 2'-H, 6'-H), 7.27 (2H, d, J = 8.4 Hz, 3'-H, 5'-H), 7.20 (1H, dd, J = 5.2 Hz, 8.8 Hz, 7-H), 7.00 (1H, s, 10-H), 6.42 (1H, td, J = 2.4 Hz, 9.2 Hz, 6-H), 6.27 (1H, dd, J = 2.4 Hz, 9.2 Hz, 4-H), 4.64 (1H, t, J = 5.6 Hz, NH), 4.04 (2H, d, J = 5.6 Hz, 3-CH_2), 2.54 (3H, s, -SCH_3), 1.95 (3H, s, 2-CH_3). HRMS calcd for $[C_{29}H_{24}FNO_2S_2+Na]^+$: 524.11247, Found: 524.11241.

3.32. (Z)-N-((5-Fluoro-2-Methyl-1-(4-(Methylthio) Benzylidene)-1H-Inden-3-yl)methyl) Naphthalene-2-Sulfonamide (39)

By following methods 7 and 9, the title compound **39** was obtained as a yellow solid in 88% (HPLC purity: 100%) yield. mp 152-154 °C. ^{1}H NMR ($CDCl_3$, 400 MHz): δ 8.40 (1H, d, J = 1.6 Hz, 1''-H), 7.91-7.87 (3H, m, 4''-H, 5''-H, 8''-H), 7.78 (1H, dd, J = 2.0 Hz, 8.8 Hz, 3''-H), 7.65-7.56 (3H, m, 6''-H, 7''-H), 7.26 (4H, d, J = 8.4 Hz, 2'-H, 3'-H, 5'-H, 6'-H), 7.20 (1H, dd, J = 4.8 Hz, 8.4 Hz, 7-H), 7.01 (1H, s, 10-H), 6.64 (1H, dd, J = 2.8 Hz, 9.2 Hz, 4-H), 6.48 (1H, td, J = 2.4 Hz, 9.2 Hz, 6-H), 4.55 (1H, t, J = 5.6 Hz, NH), 4.15 (2H, d, J = 5.6 Hz, 3-CH_2), 2.53 (3H, s, -SCH_3), 2.05 (3H, s, 2-CH_3). HRMS calcd for $[C_{29}H_{24}FNO_2S_2+Na]^+$: 524.11247, Found: 524.11248.

3.33.. (Z)-5-(Dimethylamino)-N-((5-Fluoro-2-Methyl-1-(4-(Methylthio) Benzylidene)-1H-Inden-3-yl) Methyl)naphthalene-1-Sulfonamide (40)

By following methods 7 and 9, the title compound **40** was obtained as a yellow solid in 83% (HPLC purity: 100%) yield. mp 173-175 °C. ^{1}H NMR ($CDCl_3$, 400 MHz): δ 8.49 (1H, dt, J = 1.2 Hz, 8.4 Hz, 2''-H), 8.25 (1H, dd, J = 1.2 Hz, 7.2 Hz, 4''-H), 8.22 (1H, d, J = 8.8 Hz, 8''-H), 7.52-7.44 (2H, m, 3''-H, 7''-H), 7.36 (2H, d, J = 8.0 Hz, 2'-H, 6'-H), 7.26 (2H, d, J = 8.4 Hz, 3'-H, 5'-H), 7.20 (1H, dd, J = 5.2 Hz, 8.4 Hz, 7-H), 7.15 (1H, dd, J = 0.8 Hz, 7.6 Hz, 6''-H), 7.01 (1H, s, 10-H), 6.41 (1H, td, J = 2.4 Hz, 8.8 Hz, 6-H), 6.26 (1H, dd, J = 2.4 Hz, 9.2 Hz, 4-H), 4.64 (1H, t, J = 5.2 Hz, NH), 4.02 (2H, d, J = 5.6 Hz, 3-CH_2), 2.86 (6H, s, 5''-$N(CH_3)_2$), 2.54 (3H, s, -SCH_3), 1.96 (3H, s, 2-CH_3). HRMS calcd for $[C_{31}H_{29}FN_2O_2S_2+H]^+$: 545.17272, Found: 545.17244.

3.34. (Z)-N-((5-Fluoro-2-Methyl-1-(3,4,5-Trimethoxybenzylidene)-1H-Inden-3-yl) Methyl)methanesulfonamide (41)

By following methods 7 and 9, the title compound **41** was obtained as a yellow solid in 68% (HPLC purity: 100%) yield. mp 116-118 °C. ^{1}H NMR ($CDCl_3$, 400 MHz): δ 7.51 (1H, dd, J = 4.8 Hz, 8.0 Hz, 7-H), 7.21 (1H, s, 10-H), 6.99 (1H, dd, J = 2.4 Hz, 9.2 Hz, 4-H), 6.75 (2H, s, 2'-H, 6'-H), 6.66 (1H, td, J = 2.4 Hz, 8.8 Hz, 6-H), 4.33-4.29 (3H, m, 3-$C\underline{H}_2N\underline{H}$), 3.93 (3H, s, 4'-$OCH_3$), 3.85 (6H, s, 3'-$OCH_3$, 5'-$OCH_3$), 2.94 (3H, s, SO_2CH_3), 2.26 (3H, s, 2-CH_3). HRMS calcd for $[C_{22}H_{24}FNO_5S+H]^+$: 434.14320, Found: 434.14311.

3.35. (Z)-N-((5-Fluoro-2-Methyl-1-(3,4,5-Trimethoxybenzylidene)-1H-Inden-3-yl) Methyl)ethanesulfonamide (42)

By following methods 7 and 9, the title compound **42** was obtained as a yellow solid in 68% (HPLC purity: 100%) yield. mp 132-134 °C. ^{1}H NMR ($CDCl_3$, 400 MHz): δ 7.49 (1H, dd, J = 4.8 Hz, 8.4 Hz, 7-H), 7.20 (1H, s, 10-H), 6.99 (1H, dd, J = 2.4 Hz, 9.2 Hz, 4-H), 6.75 (2H, s, 2'-H, 6'-H), 6.66 (1H, td, J = 2.4 Hz, 8.8 Hz, 6-H), 4.28 (2H, d, J = 5.6 Hz, 3-CH_2), 4.19 (1H, t, J = 5.6 Hz, NH), 3.93 (3H, s, 4'-OCH_3), 3.85 (6H, s, 3'-OCH_3, 5'-OCH_3), 3.04 (2H, q, J = 7.6 Hz, $SO_2C\underline{H}_2CH_3$), 2.25 (3H, s, 2-CH_3), 1.35 (2H, t, J = 7.6 Hz, $SO_2CH_2C\underline{H}_3$). HRMS calcd for $[C_{23}H_{26}FNO_5S+H]^+$: 448.15885, Found: 448.15871.

3.36. (Z)-N-((5-Fluoro-2-Methyl-1-(3,4,5-Trimethoxybenzylidene)-1H-Inden-3-yl) Methyl) Benzenesul Fonamide (43)

By following methods 7 and 9, the title compound **43** was obtained as a yellow solid in 67% (HPLC purity: 100%) yield. mp 161-163 °C. ^{1}H NMR ($CDCl_3$, 400 MHz): δ 7.91-7.50 (5H, m, Ph-H), 7.41 (1H, dd, J = 4.8 Hz, 8.0 Hz, 7-H), 7.12 (1H, s, 10-H), 6.71 (2H, s, 2'-H, 6'-H), 6.61-6.53 (2H, m, 6-H, 4-H), 4.39 (1H, t, J = 5.6 Hz, NH), 4.11 (2H, d, J = 5.6 Hz, 3-CH_2), 3.92 (3H, s, 4'-OCH_3), 3.84 (6H, s, 3'-OCH_3, 5'-OCH_3), 2.09 (3H, s, 2-CH_3). HRMS calcd for $[C_{27}H_{26}FNO_5S+Na]^+$: 518.14079, Found: 518.14040.

3.37. (Z)-N-((5-Fluoro-2-Methyl-1-(3,4,5-Trimethoxybenzylidene)-1H-Inden-3-yl) Methyl)-4-Methylbenzenesul Fonamide (44)

By following methods 7 and 9, the title compound **44** was obtained as a yellow solid in 64% (HPLC purity: 100%) yield. mp 155-157 °C. ^{1}H NMR ($CDCl_3$, 400 MHz): δ 7.79 (1H, d, J = 8.4 Hz, 2''-H, 6''-H), 7.41 (1H, dd, J = 5.2 Hz, 8.4 Hz, 7-H), 7.33 (1H, dd, J = 0.8 Hz, 7.6 Hz, 3''-H, 5''-H), 7.12 (1H, s, 10-H), 6.71 (2H, s, 2'-H, 6'-H), 6.58 (1H, td, J = 2.0 Hz, 8.8 Hz, 6-H), 6.52 (1H, dd, J = 2.4 Hz, 8.8 Hz, 4-H), 4.30 (1H, t, J = 5.6 Hz, NH), 4.08 (2H, d, J = 5.2 Hz, 3-CH_2), 3.92 (3H, s, 4'-OCH_3), 3.83 (6H, s, 3'-OCH_3, 5'-OCH_3), 2.44 (3H, s, 4''-CH_3), 2.10 (3H, s, 2-CH_3). HRMS calcd for $[C_{28}H_{28}FNO_5S+Na]^+$: 532.15644, Found: 532.15618.

3.38. (Z)-N-((5-Fluoro-2-Methyl-1-(3,4,5-Trimethoxybenzylidene)-1H-Inden-3-yl)Methyl) Naphthalene-1-Sul Fonamide (45)

By following methods 7 and 9, the title compound **45** was obtained as a yellow solid in 66% (HPLC purity: 100%) yield. mp 88-90 °C. ^{1}H NMR ($CDCl_3$, 400 MHz): δ 8.59 (1H, d, J = 8.4 Hz, 8''-H), 8.29 (1H, dd, J = 1.2 Hz, 7.6 Hz, 2''-H), 8.03 (1H, d, J = 8.4 Hz, 4''-H), 7.91 (1H, dd, J = 1.2 Hz, 8.0 Hz, 5''-H), 7.65-7.56 (2H, m, 6''-H, 7''-H), 7.51 (1H, t, J = 7.6 Hz, 3''-H), 7.30 (1H, dd, J = 5.2 Hz, 8.4 Hz, 7-H), 7.03 (1H, s, 10-H), 6.67 (2H, s, 2'-H, 6'-H), 6.46 (1H, td, J = 2.4 Hz, 8.4 Hz, 6-H), 6.29 (1H, dd, J = 2.8 Hz, 8.8 Hz, 4-H), 4.64 (1H, t, J = 5.2 Hz, NH), 4.04 (2H, d, J = 5.2 Hz, 3-CH_2), 3.92 (3H, s, 4'-OCH_3), 3.83 (6H, s, 3'-OCH_3, 5'-OCH_3), 1.96 (3H, s, 2-CH_3). HRMS calcd for $[C_{31}H_{28}FNO_5S+Na]^+$: 568.15644, Found: 568.15615.

3.39. (Z)-N-((5-Fluoro-2-Methyl-1-(3,4,5-Trimethoxybenzylidene)-1H-Inden-3-yl) Methyl)naphthalene-2-Sulfonamide (46)

By following methods 7 and 9, the title compound **46** was obtained as a yellow solid in 69% (HPLC purity: 97.4%) yield. mp 118-120 °C. ^{1}H NMR ($CDCl_3$, 400 MHz): δ 8.46 (1H, d, J = 2.0 Hz, 2''-H), 7.96-7.89 (3H, m, 4''-H, 5''-H, 8''-H), 7.82 (1H, dd, J = 1.6 Hz, 8.4 Hz, 3''-H), 7.67-7.58 (2H, m, 6''-H, 7''-H), 7.34 (1H, dd, J = 5.2 Hz, 8.4 Hz, 7-H), 7.05 (1H, s, 10-H), 6.66 (1H, dd, J = 2.4 Hz, 8.8 Hz, 4-H), 6.65 (2H, s, 2'-H, 6'-H), 6.52 (1H, td, J = 2.8 Hz, 8.8 Hz, 6-H), 4.52 (1H, t, J = 5.2 Hz, NH), 4.14 (2H, d, J = 5.6 Hz, 3-CH_2), 3.92 (3H, s, 4'-OCH_3), 3.82 (6H, s, 3'-OCH_3, 5'-OCH_3), 2.06 (3H, s, 2-CH_3). HRMS calcd for $[C_{31}H_{28}FNO_5S+Na]^+$: 568.15644, Found: 568.15630.

3.40. (Z)-5-(Dimethylamino)-N-((5-Fluoro-2-Methyl-1-(3,4,5-Trimethoxybenzylidene)-1H-Inden-3-yl) Methyl) Naphthalene-1-Sulfonamide (47)

By following methods 7 and 9, the title compound **47** was obtained as a yellow solid in 72% (HPLC purity: 100%) yield. mp 92-94 °C. ^{1}H NMR ($CDCl_3$, 400 MHz): δ 8.51 (1H, dt, J = 1.2 Hz, 8.0 Hz, 2''-H), 8.27-8.23 (2H, m, 4''-H, 8''-H), 7.52 (1H, dd, J = 7.6 Hz, 8.8 Hz, 3''-H), 7.48 (1H, dd, J = 7.6 Hz, 8.8 Hz, 7''-H), 7.30 (1H, dd, J = 5.2 Hz, 8.4 Hz, 7-H), 7.17 (1H, dd, J = 0.8 Hz, 7.2 Hz, 6''-H), 7.03 (1H, s, 10-H), 6.68 (2H, s, 2'-H, 6'-H), 6.44 (1H, td, J = 2.4 Hz, 9.2 Hz, 6-H), 6.27 (1H, dd, J = 2.4 Hz, 9.2 Hz, 4-H), 4.64 (1H, t, J = 5.6 Hz, NH), 4.02 (2H, d, J = 5.6 Hz, 3-CH_2), 3.91 (3H, s, 4'-OCH_3), 3.84 (6H, s, 3'-OCH_3, 5'-OCH_3), 2.86 (6H, s, 5''-$N(CH_3)_2$), 1.99 (3H, s, 2-CH_3). HRMS calcd for $[C_{33}H_{33}FN_2O_5S+H]^+$: 589.21670, Found: 589.21656.

3.41. (Z)-N,N-Dibenzyl-1-(5-Fluoro-2-Methyl-1-(4-(Methylthio) Benzylidene)-1H-Inden-3-yl) Methanamine (48)

By following methods 7 and 4, the title compound **48** was obtained as a yellow viscous liquid in 74% (HPLC purity: 93.6%) yield. ^{1}H NMR ($CDCl_3$, 400 MHz): δ 7.41 (2H, d, J = 8.4 Hz, 2'-H, 6'-H), 7.36-7.23 (13H, m, 7-H, 3'-H, 5'-H, Ph-H), 7.05 (1H, s, 10-H), 6.99 (1H, dd, J = 2.8 Hz, 9.2 Hz, 4-H), 6.53 (1H, td, J = 2.0 Hz, 9.2 Hz, 6-H), 3.53 (4H, s, CH_2-Ph), 3.46 (2H, s, 3-CH_2), 2.53 (3H, s, -SCH_3), 2.18 (3H, s, 2-CH_3). HRMS calcd for $[C_{33}H_{30}FNS+H]^+$: 492.21558, Found: 492.21598.

3.42. (Z)-1-(5-Fluoro-2-Methyl-1-(4-(Methylthio) Benzylidene)-1H-Inden-3-yl)-N,N-Bis (Pyridin-4-Ylmethyl) Methanamine (49)

By following methods 7 and 4, the title compound **49** was obtained as a yellow viscous liquid in 84% (HPLC purity: 95.9%) yield. ^{1}H NMR ($CDCl_3$, 400 MHz): δ 8.56 (4H, dd, J = 1.6 Hz, 4.8 Hz, 2''-H, 6''-H), 7.41 (2H, d, J = 8.4 Hz, 2'-H, 6'-H), 7.32 (1H, dd, J = 5.2 Hz, 8.4 Hz, 7-H), 7.28-7.26 (6H, m, 3'-H, 5'-H, 3''-H, 5''-H), 7.09 (1H, s, 10-H), 6.98 (1H, dd, J = 2.4 Hz, 9.6 Hz, 4-H), 6.58 (1H, td, J = 2.4 Hz, 9.2 Hz, 6-H), 3.55 (4H, s, CH_2-Ar), 3.51 (2H, s, 3-CH_2), 2.53 (3H, s, -SCH_3), 2.18 (3H, s, 2-CH_3). HRMS calcd for $[C_{31}H_{28}FN_3S+H]^+$: 494.20607, Found: 494.20636.

3.43. (Z)-1-(5-Fluoro-2-Methyl-1-(4-(Methylthio) Benzylidene)-1H-Inden-3-yl)-N,N-Bis(4-Fluorobenzyl) Methanamine (50)

By following methods 7 and 4, the title compound **50** was obtained as a yellow viscous liquid in 81% (HPLC purity: 100%) yield. ^{1}H NMR ($CDCl_3$, 400 MHz): δ 7.41 (2H, d, J = 8.0 Hz, 2'-H, 6'-H), 7.30 (1H, dd, J = 5.6 Hz, 8.8 Hz, 7-H), 7.28-7.25 (6H, m, 3'-H, 5'-H, 2''-H, 6''-H), 7.06 (1H, s, 10-H), 7.02-6.90 (5H, m, 4-H, 3''-H, 5''-H), 6.55 (1H, td, J = 2.8 Hz, 9.2 Hz, 6-H), 3.47 (4H, s, CH_2-Ar), 3.44 (2H, s, 3-CH_2), 2.53 (3H, s, -SCH_3), 2.17 (3H, s, 2-CH_3). HRMS calcd for $[C_{33}H_{28}F_3NS+H]^+$: 528.19673, Found: 528.19714.

3.44. (Z)-4-(((4-(Dimethylamino) Benzyl)((5-Fluoro-2-methyl-1-(4-(Methylthio) Benzylidene)-1H-Inden-3-yl) Methyl)Amino) Methyl)-N,N-Dimethylaniline (51)

By following methods 7 and 4, the title compound **51** was obtained as a yellow solid in 82% (HPLC purity: 100%) yield. mp 69-71 °C. ^{1}H NMR ($CDCl_3$, 400 MHz): δ 7.41 (2H, d, J = 8.0 Hz, 2'-H, 6'-H), 7.28-7.18 (7H, m, 7-H, 3'-H, 5'-H, 2''-H, 6''-H), 7.05 (1H, dd, J = 2.4 Hz, 9.2 Hz, 4-H), 7.02 (1H, s, 10-H), 6.72-6.68 (4H, m, 3''-H, 5''-H), 6.51 (1H, td, J = 2.8 Hz, 9.2 Hz, 6-H), 3.42 (4H, s, CH_2-Ar, 3-CH_2), 2.91 (12H, s, 4''-$N(CH_3)_2$), 2.53 (3H, s, -SCH_3), 2.16 (3H, s, 2-CH_3). HRMS calcd for $[C_{37}H_{40}FN_3S+H]^+$: 578.29997, Found: 578.30009.

3.45. (Z)-1-(5-Fluoro-2-methyl-1-(4-(Methylthio) Benzylidene)-1H-Inden-3-yl)-N,N-bis (Naphthalen-2-Ylmethyl) Methanamine (52)

By following methods 7 and 4, the title compound **52** was obtained as a yellow solid in 88% (HPLC purity: 100%) yield. mp 81-83 °C. ^{1}H NMR ($CDCl_3$, 400 MHz): δ 7.83-7.77 (8H, m, 1''-H, 4''-H, 5''-H, 8''-H), 7.52 (2H, dd, J = 1.6 Hz, 8.8 Hz, 3''-H), 7.48-7.41 (4H, m, 6''-H, 7''-H), 7.36 (2H, d, J = 8.0 Hz, 2'-H, 6'-H), 7.27-7.23 (3H, m, 7-H, 3'-H, 5'-H), 7.08 (1H, dd, J = 2.4 Hz, 9.6 Hz, 4-H), 7.03 (1H, s, 10-H), 6.53 (1H, td, J = 2.4 Hz, 9.2 Hz, 6-H), 3.73 (4H, s, CH_2-Ar), 3.56 (2H, s, 3-CH_2), 2.52 (3H, s, -SCH_3), 2.20 (3H, s, 2-CH_3). HRMS calcd for $[C_{41}H_{34}FNS+H]^+$: 592.24688, Found: 592.24759.

3.46. (Z)-N-((5-Fluoro-2-Methyl-1-(4-(Methylthio)benzylidene)-1H-Inden-3-yl) Methyl)-N-Phenethyl-2-Phenylethanamine (53)

By following methods 7 and 4, the title compound **53** was obtained as a yellow solid in 83% (HPLC purity: 100%) yield. mp 78-80 °C. ^{1}H NMR ($CDCl_3$, 400 MHz): δ 7.45 (2H, d, J = 8.0 Hz, 2'-H, 6'-H), 7.32-7.11 (13H, m, 7-H, 3'-H, 5'-H, Ph-H), 7.07 (1H, s, 10-H), 7.00 (1H, dd, J = 2.4 Hz, 8.8 Hz, 4-H), 6.53 (1H, td, J = 2.4 Hz, 9.2 Hz, 6-H), 3.66 (2H, s, 3-CH_2), 2.81 (8H, s, CH_2CH_2-Ph), 2.54 (3H, s, -SCH_3), 2.17 (3H, s, 2-CH_3). HRMS calcd for $[C_{35}H_{34}FNS+H]^+$: 520.24688, Found: 520.24683.

3.47. (Z)-1-(5-Fluoro-2-Methyl-1-(4-(Methylthio)benzylidene)-1H-Inden-3-yl)-N,N-Bis(Furan-2-Ylmethyl) Methanamine (54)

By following methods 7 and 4, the title compound **54** was obtained as a yellow viscous liquid in 87% (HPLC purity: 100%) yield. ^{1}H NMR ($CDCl_3$, 400 MHz): δ 7.44 (2H, d, J = 8.4 Hz, 2'-H, 6'-H), 7.42 (2H, dd, J = 0.8 Hz, 2.0 Hz, 5''-H), 7.31 (1H, dd, J = 5.2 Hz, 8.4 Hz, 7-H), 7.28 (2H, d, J = 8.4 Hz, 3'-H, 5'-H), 7.11 (1H, dd, J = 2.4 Hz, 9.6 Hz, 4-H), 7.07 (1H, s, 10-H), 6.54 (1H, td, J = 2.4 Hz, 8.8 Hz, 6-H), 6.35 (2H, dd, J = 1.6 Hz, 3.2 Hz, 4''-H), 6.23 (2H, d, J = 2.8 Hz, 3''-H), 3.69 (4H, s, CH_2-Ar), 3.57 (2H, s, 3-CH_2), 2.54 (3H, s, -SCH_3), 2.17 (3H, s, 2-CH_3). HRMS calcd for $[C_{29}H_{26}FNO_2S+H]^+$: 472.17410, Found: 472.17452.

3.48. (Z)-N,N-Dibenzyl-1-(5-Fluoro-2-Methyl-1-(3,4,5-Trimethoxybenzylidene)-1H-Inden-3-yl) Methanamine (55)

By following methods 7 and 4, the title compound **55** was obtained as a yellow solid in 69% (HPLC purity: 96.6%) yield. mp 87-88 °C. ^{1}H NMR ($CDCl_3$, 400 MHz): δ 7.37-7.22 (11H, m, 7-H, Ph-H), 7.05 (1H, s, 10-H), 7.00 (1H, dd, J = 2.8 Hz, 9.6 Hz, 4-H), 6.72 (2H, s, 2'-H, 6'-H), 6.56 (1H, td, J = 2.8 Hz, 8.8 Hz, 6-H), 3.91 (3H, s, 4'-OCH_3), 3.83 (6H, s, 3'-OCH_3, 5'-OCH_3), 3.54 (4H, s, CH_2Ph), 3.47 (2H, s, 3-CH_2), 2.18 (3H, s, 2-CH_3). HRMS calcd for $[C_{35}H_{34}FNO_3+H]^+$: 536.25955, Found: 536.25931.

3.49. (Z)-1-(5-Fluoro-2-Methyl-1-(3,4,5-Trimethoxybenzylidene)-1H-Inden-3-yl)-N,N-Bis (Pyridin-4-ylmethyl) Methanamine (56)

By following methods 7 and 4, the title compound **56** was obtained as a yellow solid in 75% (HPLC purity: 100%) yield. mp 65-67 °C. ^{1}H NMR ($CDCl_3$, 400 MHz): δ 8.56 (4H, dd, J = 1.6 Hz, 4.4 Hz, 2''-H, 6''-H), 7.39 (1H, dd, J = 5.2 Hz, 8.4 Hz, 7-H), 7.28 (4H, dd, J = 1.6 Hz, 4.8 Hz, 3''-H, 5''-H), 7.09 (1H, s, 10-H), 7.00 (1H, dd, J = 2.0 Hz, 9.2 Hz, 4-H), 6.71 (2H, s, 2'-H, 6'-H), 6.60 (1H, td, J = 2.4 Hz, 9.2 Hz, 6-H), 3.92 (3H, s, 4'-OCH_3), 3.83 (6H, s, 3'-OCH_3, 5'-OCH_3), 3.56 (4H, s, CH_2Ar), 3.51 (2H, s, 3-CH_2), 2.19 (3H, s, 2-CH_3). HRMS calcd for $[C_{33}H_{32}FN_3O_3+H]^+$: 538.25005, Found: 538.25017.

3.50. (Z)-1-(5-Fluoro-2-Methyl-1-(3,4,5-Trimethoxybenzylidene)-1H-Inden-3-yl)-N,N-Bis(4-Fluorobenzyl) Methanamine (57)

By following methods 7 and 4, the title compound **57** was obtained as a yellow viscous liquid in 68% (HPLC purity: 100%) yield. ^{1}H NMR ($CDCl_3$, 400 MHz): δ 7.36 (1H, dd, J = 4.8 Hz, 8.0 Hz, 7-H), 7.30-7.26 (4H, m, 2''-H, 6''-H), 7.06 (1H, s, 10-H), 7.03-6.98 (4H, m, 3''-H, 5''-H), 6.95 (1H, dd, J = 2.8 Hz, 9.6 Hz, 4-H), 6.71 (2H, s, 2'-H, 6'-H), 6.57 (1H, td, J = 2.8 Hz, 9.2 Hz, 6-H), 3.91 (3H, s, 4'-OCH_3), 3.83 (6H, s, 3'-OCH_3, 5'-OCH_3), 3.48 (4H, s, CH_2Ar), 3.45 (2H, s, 3-CH_2), 2.17 (3H, s, 2-CH_3). HRMS calcd for $[C_{35}H_{32}F_3NO_3+H]^+$: 572.24070, Found: 572.24037.

3.51. (Z)-1-(5-Fluoro-2-Methyl-1-(3,4,5-Trimethoxybenzylidene)-1H-Inden-3-yl)-N,N-Bis (Naphthalen-2-Ylmethyl) Methanamine (58)

By following methods 7 and 4, the title compound **58** was obtained as a yellow solid in 69% (HPLC purity: 95.2%) yield. mp 95-97 °C. ^{1}H NMR ($CDCl_3$, 400 MHz): δ 7.85-7.78 (8H, m, 1''-H, 4''-H, 5''-H, 8''-H), 7.54 (2H, dd, J = 1.2 Hz, 8.4 Hz, 3''-H), 7.49-7.42 (4H, m, 6''-H, 7''-H), 7.35 (1H, dd, J = 5.6 Hz, 8.8 Hz, 7-H), 7.10 (1H, dd, J = 2.4 Hz, 9.2 Hz, 4-H), 7.06 (1H, s, 10-H), 6.70 (2H, s, 2'-H, 6'-H), 6.56 (1H, td, J = 2.4 Hz, 9.2 Hz, 6-H), 3.91 (3H, s, 4'-OCH_3), 3.82 (6H, s, 3'-OCH_3, 5'-OCH_3), 3.73 (4H, s, CH_2Ar), 3.56 (2H, s, 3-CH_2), 2.21 (3H, s, 2-CH_3). HRMS calcd for $[C_{43}H_{38}FNO_3+H]^+$: 636.29085, Found: 636.29011.

3.52. (Z)-1-(5-Fluoro-2-Methyl-1-(3,4,5-Trimethoxybenzylidene)-1H-Inden-3-yl)-N,N-Bis(4-methoxybenzyl) Methanamine (59)

By following methods 7 and 4, the title compound **59** was obtained as a yellow solid in 68% (HPLC purity: 97.6%) yield. mp 89-91 °C. ^{1}H NMR ($CDCl_3$, 400 MHz): δ 7.34 (1H, dd, J = 5.6 Hz, 8.4 Hz, 7-H), 7.26-7.23 (4H, m, 2''-H, 6''-H), 7.05 (1H, s, 10-H), 6.99 (1H, dd, J = 2.8 Hz, 9.6 Hz, 4-H), 6.87-6.83 (4H, m, 3''-H, 5''-H), 6.72 (2H, s, 2'-H, 6'-H), 6.55 (1H, td, J = 2.4 Hz, 8.8 Hz, 6-H), 3.91 (3H, s, 4'-OCH_3), 3.83 (6H, s, 3'-OCH_3, 5'-OCH_3), 3.79 (6H, s, 4''-OCH_3), 3.45 (4H, s, CH_2Ar), 3.43 (2H, s, 3-CH_2), 2.17 (3H, s, 2-CH_3). HRMS calcd for $[C_{37}H_{38}FNO_5+H]^+$: 596.28068, Found: 596.28034.

3.53. (Z)-N-((5-Fluoro-2-Methyl-1-(3,4,5-Trimethoxybenzylidene)-1H-Inden-3-yl) Methyl)-N-phenethyl-2-Phenylethanamine (60)

By following methods 7 and 4, the title compound **60** was obtained as a yellow viscous liquid in 70% (HPLC purity: 94.0%) yield. ^{1}H NMR ($CDCl_3$, 400 MHz): δ 7.39 (1H, dd, J = 5.6 Hz, 8.8 Hz, 7-H), 7.27-7.12 (10H, m, Ph-H), 7.08 (1H, s, 10-H), 7.02 (1H, dd, J = 2.4 Hz, 9.2 Hz, 4-H), 6.75 (2H, s, 2'-H, 6'-H), 6.55 (1H, td, J = 2.8 Hz, 9.2 Hz, 6-H), 3.93 (3H, s, 4'-OCH_3), 3.85 (6H, s, 3'-OCH_3, 5'-OCH_3), 3.67 (2H, s, 3-CH_2), 2.82 (8H, s, CH_2CH_2Ph), 2.18 (3H, s, 2-CH_3). HRMS calcd for $[C_{37}H_{38}FNO_3+H]^+$: 564.29085, Found: 564.29065.

3.54. (Z)-1-(5-Fluoro-2-Methyl-1-(3,4,5-Trimethoxybenzylidene)-1H-inden-3-yl)-N,N-Bis (Furan-2-Ylmethyl) Methanamine (61)

By following methods 7 and 4, the title compound **61** was obtained as a yellow viscous liquid in 72% (HPLC purity: 94.0%) yield. ^{1}H NMR ($CDCl_3$, 400 MHz): δ 7.43 (1H, dd, J = 0.8 Hz, 2.0 Hz, 5''-H), 7.38 (1H, dd, J = 5.6 Hz, 8.4 Hz, 7-H), 7.11 (1H, dd, J = 2.4 Hz, 9.6 Hz, 4-H), 7.08 (1H, s, 10-H), 6.74 (2H, s, 2'-H, 6'-H), 6.57 (1H, td, J = 2.8 Hz, 8.8 Hz, 6-H), 6.35 (1H, dd, J = 2.0 Hz, 3.6 Hz, 4''-H), 6.24 (1H, dd, J = 0.8 Hz, 3.6 Hz, 3''-H), 3.92 (3H, s, 4'-OCH_3), 3.84 (6H, s, 3'-OCH_3, 5'-OCH_3), 3.69 (4H, s, CH_2Fu), 3.58 (2H, s, 3-CH_2), 2.18 (3H, s, 2-CH_3). HRMS calcd for $[C_{31}H_{30}FNO_5+H]^+$: 516.21808, Found: 516.21753.

© 2018 Mathew *et al*.

This is an open access article distributed under the terms of the Creative Commons Attribution 4. 0 International Public License (CC-BY 4. 0), a copy of which is available at: https://creativecommons. org/licenses/by/4. 0/legalcode. This license permits unrestricted use, distribution, and reproduction in any medium, provided the original author and source are credited.
